# MicroR408 regulates defense response upon wounding in sweet potato

**DOI:** 10.1093/jxb/ery381

**Published:** 2018-11-05

**Authors:** Yun-Wei Kuo, Jeng-Shane Lin, Yu-Chi Li, Min-Yao Jhu, Yu-Chi King, Shih-Tong Jeng

**Affiliations:** 1 Institute of Plant Biology and Department of Life Science, National Taiwan University, Taipei, Taiwan; 2 Department of Life Sciences, National Chung Hsing University, Taichung, Taiwan

**Keywords:** *GAUT*, *KCS*, miR408, *PCL*, small RNA deep sequencing, sweet potato, wounding

## Abstract

MiRNAs play diverse roles in plant development and defense responses by binding to their mRNA targets based on sequence complementarity. Here, we investigated a wound-related miR408 and its target genes in sweet potato (*Ipomoea batatas*) by small RNA deep sequencing and transcriptome analysis. The expression patterns of miR408 and the *miR408 precursor* were significantly repressed by wounding and jasmonate (JA). In contrast, expression of the putative target genes *IbKCS* (3-ketoacyl-CoA synthase 4), *IbPCL* (plantacyanin), and *IbGAUT* (galacturonosyltransferase 7-like) of miR408 was increased following wounding, whereas only *IbKCS* was increased after JA treatment. Target cleavage site mapping and *Agrobacterium*-mediated transient assay demonstrated that *IbKCS, IbPCL*, and *IbGAUT* were the targets of miR408. The expression of miR408 target genes was repressed in transgenic sweet potatoes overexpressing miR408. These data indicated a relationship between miR408 and its target genes. Notably, miR408-overexpressing plants showed a semi-dwarf phenotype and attenuated resistance to insect feeding, while transgenic plants overexpressing *IbKCS* exhibited more insect resistance than plants overexpressing only the empty vector. Collectively, sweet potato reduces the abundance of miR408 upon wounding to elevate the expression of *IbKCS*, *IbPCL*, and *IbGAUT*. The expression of *IbKCS* enhances the defense system against herbivore wounding.

## Teaser

Repression of miR408 activates the expression of three miR408 target genes, *IbKCS*, *IbPCL*, and *IbGAUT*, leading to activation of defense response against wounding in sweet potato.

## Introduction

Plants possess complex signaling systems to survive biotic and abiotic stressors (Wong and Shimamoto, 2009). Unlike animals who can escape from extreme environments, plants respond to different stresses by activating the expression of various genes. Wounding is one of the frequent stresses for terrestrial plants (Savatin *et al.*, 2014). The physical damage caused by rain, wind, herbivore feeding, or microbial attack triggers wounding responses in plants (de Bruxelles and Roberts, 2001), and these responses affect plant growth and development (Bowles, 1990; Savatin *et al.*, 2014). Plants have evolved complex defense systems against wounding. Responses to damage are local, systemic, or both (León *et al.*, 2001). Early wounding signals, including jasmonate (JA), ethylene, Ca^2+^ influx, nitric oxide, and H_2_O_2_, can regulate wounding-responsive genes to function in defense-related processes (Pozo *et al.*, 2008; Wasternack and Hause, 2013). In response to a wounding signal from herbivory, plants activate the expression of proteinase inhibitor and lectin genes, which impair the activity of insect digestive enzymes to decrease the damage by herbivory (Howe, 2004; Chen *et al.*, 2008; Chen *et al.*, 2016*b*). In addition, several factors, including lignin, pectin, suberin, and waxes, are stimulated in wounded tissue to heal the tissue or to form a physical barrier to prevent further damage (Eigenbrode and Espelie, 1995; Kunst and Samuels, 2003; Pollard *et al.*, 2008; War *et al.*, 2012)

MiRNAs are important regulators in developmental processes and stress responses in plants (Jones-Rhoades and Bartel, 2004; Voinnet, 2009; Rogers and Chen, 2013; Budak *et al.*, 2015; Niu *et al.*, 2016). MiRNAs are usually 20–24 nucleotides in length (Zhang *et al.*, 2006; Rogers and Chen, 2013), and are evolutionarily conserved (Lau *et al.*, 2001; Fukao *et al.*, 2007). These non-coding miRNAs are processed from the single-stranded endogenous transcripts, which fold into hairpin stem–loop structures called primary miRNAs (pri-miRNAs) (Sunkar *et al.*, 2007; Mendes *et al.*, 2009; Miskiewicz *et al.*, 2017). Pri-miRNAs are cleaved by DICER-LIKE 1 (DCL1) to form precursor miRNA (pre-miRNA) (Kim, 2005; Rogers and Chen, 2013). DCL1 also carries out the cleavage of pre-miRNA to release the short miRNA:miRNA* duplex (Axtell *et al.*, 2011), in which miRNA is the guide strand and miRNA* is the degraded strand (Voinnet, 2009; Samad *et al.*, 2017). The guide strand miRNAs incorporate into the RNA-induced silencing complex (RISC) through binding with Argonaute (AGO) proteins (Budak and Akpinar, 2015). The mature miRNAs target nearly perfect complementary sequences (Reinhart *et al.*, 2002; Rhoades *et al.*, 2002) to cleave their target mRNA, resulting in transcriptional gene silencing or translational inhibition (Bartel, 2004; Khraiwesh *et al.*, 2010; Ameres and Zamore, 2013; Rogers and Chen, 2013). Plant miRNAs have been demonstrated to regulate the expression of genes encoding transcription factors, stress-responsive proteins, and growth-related proteins (Rhoades *et al.*, 2002; Jones-Rhoades and Bartel, 2004; Voinnet, 2009; Li and Zhang, 2016; Samad *et al.*, 2017). These conserved miRNAs have multiple functions in different plants against environmental factors, including oxidative stress, copper or phosphate deficiency, ultraviolet stress, salt stress, abscisic acid stress, water deficiency, osmotic stress, and pathogen infection (Reinhart *et al.*, 2002; Abdel-Ghany and Pilon, 2008; Jagadeeswaran *et al.*, 2009; Jia *et al.*, 2009a, b; Yamasaki *et al.*, 2009; Zhu *et al.*, 2011; Juszczak and Baier, 2012).

Several miRNAs involved in wounding have been identified (Bozorov *et al.*, 2012; Lin *et al.*, 2012, 2013; Tang *et al.*, 2012; Wang *et al.*, 2014). The expression levels of miR159, miR160, miR167, miR396, miR403, miR408, and miR828 are increased in tobacco during wounding (Bozorov *et al.*, 2012), and those of miR156, miR164, miR166, miR168, miR171, miR172, miR319, miR390, miR393, miR394, and miR398 are enhanced particularly when herbivores attack (Bozorov *et al.*, 2012). Additionally, tobacco miR319, miR394, and miR828 are JA-dependent miRNAs, which were predicted to regulate JA biosynthesis in the face of herbivore wounding (Bozorov *et al.*, 2012). Furthermore, miR828 and small RNA8105 are induced in sweet potato upon wounding, and enhance the production of lignin to strengthen the cell wall (Lin *et al.*, 2012, 2013). This sophisticated regulatory pathway of miRNA has diverse roles in plant development, hormone regulation, metabolite synthesis, and defense responses.

Sweet potato is one of the most important food crops with high resistance to stress, especially wounding. Several researchers indicated that multiple transcriptional regulations are activated against wounding in sweet potato (Lin et al., 2012, 2013, 2014; Chen *et al.*, 2016a, b; Li *et al.*, 2016). In this study, we further investigate the wound-repressible miRNAs in sweet potato. Small RNA deep sequencing was performed to survey the wound-responsive miRNAs extensively. Analytical and functional characterizations of these wound-related miRNAs, miRNA precursors, and their target genes allow us to determine the miRNA-mediated mechanisms in sweet potato upon wounding.

## Materials and methods

### Plant materials, growth conditions, and stress treatments

Sweet potato (*Ipomoea batatas* cv. Tainung 57) and tobacco (*Nicotiana tabacum* L. cv. W38) plants were grown in growth chambers (16 h/25 °C light and 8 h/22 °C dark; 70% humidity) under illumination of 30 µmol photons m^–2^ s^–1^ and 60 µmol photons m^–2^ s^–1^, respectively. Plants with 6–8 fully developed leaves were used in this study. The third fully expanded sweet potato leaves counted from the terminal bud were treated by wounding, 50 µM methyl jasmonate (MeJA; Sigma, St. Louis, MO, USA), or *Spodoptera litura*. In the wounding treatment, leaves except the primary veins were pressed by tweezers. In the *S. litura* feeding assay, the third fully expanded leaves were placed in plastic Petri dishes (90 mm) containing wet filter paper. The third-instar *S. litura* were individually placed on each leaf at 25 °C under a 16 h light/8 h dark photoperiod. All the analyses were performed in at least three independent biological replicates.

### Small RNA library construction, sequencing, and processing

Total RNAs from leaves wounded for 30 min and unwounded leaves were extracted by Trizol reagent (Invitrogen, Carlsbad, CA, USA). The small RNA libraries were prepared following the Small RNA Sample Preparation Protocol of Illumina TruSeq, and then sequenced by the Illumina Genome Analyzer IIx. Sequencing data were compared with the known plant miRNAs in the miRBase database (Kozomara and Griffiths-Jones, 2011). The perfectly matching sequences were considered as the conserved miRNAs. The miRNAs whose expression levels changed were classified as wound-inducible miRNAs (>1.2-fold) and wound-repressible miRNAs (<0.8-fold). The raw data of small RNA deep sequencings were uploaded to the Gene Expression Omnibus (http://www.ncbi.nlm.nih.gov/geo) with accession number GSE115176. The miRNAs repressed in sweet potato upon wounding are shown in Supplementary Table S1 at *JXB* online.

### Transcriptome sequencing and *de novo* assembly

Total RNAs from sweet potato leaves were extracted using Trizol reagent (Invitrogen). Then, the RNA Sample Prep v2 LS Protocol of Illumina TruSeq was followed to prepare the sweet potato *de novo* transcriptome library. The transcriptome library was then sequenced by the Illumina MiSeq platform. RNA sequence contigs were *de novo* assembled using the Trinity platform (Grabherr *et al.*, 2011) (http://trinityrnaseq.sourceforge.net/). The contigs were blasted based on the Blastn/Blastx NCBI database for gene annotation.

### Prediction and validation of miR408 precursor and target genes

The small RNA deep sequencing databases were mapped with the sweet potato transcriptome database by Bowtie software (Langmead, 2010) (http://bowtie-bio.sourceforge.net/manual.shtml) to predict the wound-regulated miRNA precursors and target genes. For miR408 precursor prediction, the mRNA contigs that perfectly match with the miR408 sequence were considered as potential miR408 precursors (pre408). The secondary structure of the potential pre408 contigs was predicted by Mfold (Zuker, 2003) (http://unafold.rna.albany.edu/?q=mfold). The contig with the correct stem–loop structure forming a miRNA:miRNA* duplex was selected as pre408. Furthermore, to define and characterize *pre408*, it was isolated from sweet potato cDNA by PCR with the primer pair Pre408-F/Pre408-R (primers are listed in Supplementary Table S2).

Plant miRNAs recognize target mRNAs through sequence complementarity. To evaluate the complementarities of miRNAs and targets, the penalty scores were calculated based on the procedure described by Meyers *et al.* (2008) and Liu *et al.* (2014). The penalty scores represent the pairing between miRNAs and targets. A mismatch is defined as 1 point; a GU wobble is defined as 0.5 point; and a gap is defined as 2 points. To identify potential miR408 target genes in sweet potato, the miR408 sequence was searched against the transcriptome contig data set. The contigs that have more than three points or have a mismatch in the central region, the 9–11 nucleotides from the 5' end of miR408, were excluded from analysis.

### Small RNA blot assay

Total RNA (20 µg) extracted from sweet potato leaves was separated by electrophoresis using a 12% polyacrylamide gel containing 8 M urea (Amresco Inc., USA). Then the RNA gel was transferred to a Hybond-NX membrane (GE Healthcare, USA) and cross-linked by UV (Pall *et al.*, 2007). For miR408 detection, the blotted membranes were hybridized with the radiolabeled gene-specific RNA probes, produced by *in vitro* transcription (Jeng *et al.*, 1990, 1992) using T3 RNA polymerase (Promega, Madison, WI, USA). The antisense sequence of miR408 fused with the T3 promoter was synthesized and annealed with the T3 top strand (Supplementary Table S2) as the DNA template for transcription to synthesize the miR408 RNA probe by T3 RNA polymerase (Promega). The procedures of pre-hybridization, hybridization, and washing were performed as previously described (Lin *et al.*, 2012). The membrane was exposed to a Phosphorimager screen (Molecular Dynamics) for 3–4 d after washing, and then was scanned by Phosphorimager (Typhoon 9400). In addition, the membrane was stripped and re-hybridized with the radiolabeled 5.8S rRNA probe, produced by PCR with primers 5.8S rRNA-F/5.8S rRNA-R (Supplementary Table S2), and it served as an internal control for small RNA blot assays. Three independent experiments were performed for each sample.

### RNA extraction and quantitative real-time PCR (qRT-PCR) analysis

Total RNA was extracted from sweet potato leaves using Trizol reagent (Invitrogen), and then treated with RNase-free Turbo DNase (Thermo Fisher) to remove contaminated genomic DNA. RNA (2 µg) was used to synthesize the first-strand cDNA using Moloney murine leukemia virus (MMLV) reverse transcriptase (Invitrogen) with primer T25VN (Supplementary Table S2). The cDNAs were further analyzed by qRT-PCR (SYBR FAST qRT-PCR Master Mix, BioRad). The *IbActin* gene was used as an internal control to normalize the gene expression.

To detect the expression of miRNA, poly(A) tailing assay was performed (Shi and Chiang, 2005; C. Wang *et al.*, 2013). RNAs were polyadenylated by poly(A) polymerase (New England BioLabs) at 37 °C for 60 min. The poly(A) RNA was reverse transcribed with a poly(T) adaptor containing universal primer-1 (MatureRT-1) (Supplementary Table S2) into cDNA. For miRNA analysis, the miR408-specific primer and miRNA universal primer-1 (UniPCR 1) (Supplementary Table S2) were used for qRT-PCR (Shi and Chiang, 2005). The expression of 5.8S rRNA was used for qRT-PCR normalization (Supplementary Table S2). Three independent experiments were performed for each sample.

### Isolation and sequence characterization of *IbKCS, IbPCL*, and *IbGAUT* genes

The transcriptome data of sweet potato provide only partial gene sequences. Therefore, 5' and 3' RACE was used to obtain the full coding sequences of the targets of miRNAs. RACE was performed as previously described (Lin *et al.*, 2012). RNAs were ligated with 5-RNA adaptor and 3-RT adaptor. After being reverse transcribed to cDNA, these PCR fragments were cloned and sequenced by 5- or 3-adaptor primers and gene-specific RACE primers (Supplementary Table S2).

### Mapping of miR408 cleavage sites

Modiﬁed RNA ligase-mediated 5' RACE (5'-RLM-RACE) and 3' poly(A) polymerase-mediated RACE (3'-PPM-RACE) were used to confirm the miR408-directed cleavage site as previously described (Kasschau *et al.*, 2003; Lin *et al.*, 2012; C. Wang *et al.*, 2013). For 5'-RLM-RACE, total RNAs from sweet potato leaves were ligated to a 5-RNA adaptor (Supplementary Table S2) by T4 RNA ligase (New England Biolabs). For 3'-PPM-RACE, RNAs were polyadenylated by poly(A) polymerase (New England BioLabs), and the poly(A) RNAs were reverse transcribed with a poly(T) 3-RT adaptopr (Supplementary Table S2) into cDNA. Then, PCRs using 5- or 3-adaptor primers and gene-specific primers (Supplementary Table S2) were applied. The PCR amplicons were cloned, and sequenced to determine the cleavage sites in target mRNAs.

### Constructs and plant transformation

*Pre408* was cloned into the binary vector pCAMBIA2300 driven by the 35S promoter. These constructions were transferred into *Agrobacterium rhizogenes* strain 15834 for plant transformation. For sweet potato transformation, the leaves from virus-free tissue culture were infected by *A. rhizogenes* strain 15834 for hairy root induction (Lin *et al.*, 2012, 2013; Li *et al.*, 2016). The induced roots were further selected by 30 ppm kanamycin for 14 d. Plants regenerated from the transgenic hairy roots were used.

For ectopic expression of *IbKCS*, *IbPCL*, and *IbGAUT* in tobacco, the ORFs of these genes were cloned into the binary vector pCAMBIA2300, which harbors a 35S promoter. These constructions were then separately transferred into *Agrobacterium tumefaciens* strain LBA4404 (Horsch *et al.*, 1985). For tobacco transformation, leaf discs co-incubated with *A. tumefaciens* strain LBA4404 were used to generate transgenic plants under kanamycin selection.

### *Agrobacterium*-mediated transient expression in tobacco

*Agrobacterium tumefaciens* (LBA4404) was infiltrated into *N. tabacum* leaves as previously described (Kim *et al.*, 2009; Lin *et al.*, 2012). The sequence of *pre408* was obtained by PCR with primer set Pre408-F/Pre408-R (Supplementary Table S2), and then inserted into the pCAMBIA1300 vector. Short tandem target mimic (STTM) is an approach for silencing speciﬁc small RNAs *in vivo*. The fragment STTM408 was designed and obtained as previously described (Yan *et al.*, 2012) by PCR using primer set Xba1-STTM-Mimic408-1F/Xma1-STTM-Mimic408-2R (Supplementary Table S2), and cloned into pCAMBIA1300. The ORFs of *IbKCS*, *IbPCL*, and *IbGAUT* were amplified by PCR with gene-specific primer pairs (Supplementary Table S2), and were inserted in pCAMBIA2300.

Agrobacteria carrying pCAMBIA1300 (EV), pCAMBIA1300-*pre408* (Pre408), pCAMBIA1300-*STTM408* (STTM), pCAMBIA2300-*IbKCS* (*IbKCS*), pCAMBIA2300-*IbPCL* (*IbPCL*), or pCAMBIA2300-*IbGAUT* (*IbGAUT*) were generated to infiltrate mature leaves of tobacco. After 4 d of infiltration, total RNAs of the infiltrated leaves were isolated using Trizol reagent (Invitrogen). Total RNAs were treated with RNase-free Turbo DNase (Thermo Fisher), and then reverse transcribed to cDNAs for gene expression analyses. qRT-PCR was used to detect the expression of *IbKCS*, *IbPCL*, *IbGAUT*, *NPTII*, and *NtActin* in tobacco.

### Insect bioassay with *Spodoptera litura*

The method of insect bioassay in this study was modified from previous reports (Song *et al.*, 2013; Chen *et al.*, 2016*b*). The third fully expanded leaves of wild-type, transgenic sweet potatoes, and transgenic tobacco were placed in plastic Petri dishes (90 mm) containing wet filter paper. The second-instar *S. litura* larvae were placed on each leaf at 25 °C under a16 h light/8 h dark photoperiod. Larval weights (*n*≥10) were determined after 5, 7, and 9 d of feeding. These analyses included three independent biological repeats.

### Chlorophyll content and chlorophyll fluorescence measurement

For chlorophyll content assay, the third leaves of wild-type and transgenic sweet potatoes were extracted by 80% acetone. The leaves were then incubated in 80% acetone at 4 °C overnight, and clarified by centrifugation at 14 000 *g* for 5 min. The absorbance of the supernatant was measured at wavelengths of 645 nm and 663 nm by spectrophotometry (Inﬁnite M200 plate reader) as previously described (Minocha *et al.*, 2009). These analyses included three independent biological repeats.

Photoinhibition was determined by measuring the potential quantum yield (*F*_v_/*F*_m_) of dark-adapted samples as described by Genty *et al.* (1989). The variable/maximal fluorescence ratio (*F*_v_/*F*_m_) represents the activity of PSII, and was used to assess functional damage to the plants (Artus *et al.*, 1996). Chlorophyll fluorescence was measured by a Photosynthesis Yield Analyzer (Mini-Pam, Heinz Walz GmbH, Effeltrich, Germany) (Rascher *et al.*, 2000). The wild-type and transgenic sweet potatoes grown normally for 6 weeks were dark-adapted for 30 min before chlorophyll fluorescence measurements. After 30 min of acclimation, the *F*_v_/*F*_m_ of the third fully expanded sweet potato leaves was measured by a mini PAM at a light intensity of 110 µmol photons m^–2^ s^–1^. The *F*_v_/*F*_m_ values were examined from one leaf per plant for five plants in each transgenic line. This experiment was executed in three replicates.

### Water loss rate measurements

To compare the water loss rates between wild-type and miR408-ox sweet potato, the detached leaves from 8-week-old plants were placed on weighing paper inside the same growth room at 25 °C. The weights of leaves were determined at various intervals, and the loss of fresh weight (percentage) was used to indicate the water loss rate (Shi *et al.*, 2012; Cheng *et al.*, 2013). These analyses were repeated in three biological replicates.

## Results

### Identiﬁcation of miR408 during the wounding response

**Fig. 1.**
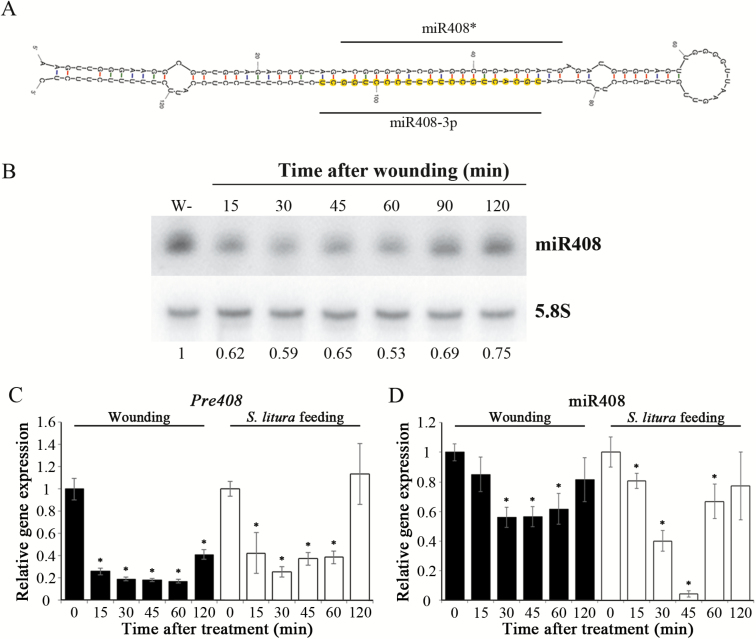
Expression patterns of *miR408 precursor* (*pre408*) and miR408 in response to wounding. (A) The secondary structure of *pre408* in sweet potato. (B) Expression patterns of miR408 in sweet potato upon wounding were analyzed by northern blotting. Total RNAs were extracted from the leaves wounded for 0 (W–), 15, 30, 45, 60, and 120 min. Northern blotting was used to determine the expression levels of miR408. The expression levels of miR408 were normalized by those of 5.8S rRNA. The expression patterns of *pre408* (C) and miR408 (D) in the leaves of sweet potato wounded by mechanical wounding or insect feeding for 0, 15, 30, 45, 60, and 120 min were analyzed by qRT-PCR. The qRT-PCR results were normalized by the expression levels of *IbActin*. Data are presented as means ±SD (*n*=4). The asterisks represent a significant difference from unwounded treatment by Student’s *t*-test (*P<0.05).

Wounding-inducible miRNAs have been found and investigated in sweet potato (Lin *et al.*, 2012, 2013). In this study, we were interested in identifying the miRNAs that are repressed by wounding. To identify the wound-repressed miRNAs in the leaves of sweet potato, small RNA deep sequencing was conducted on the unwounded and wounded leaves for 30 min (Supplementary Tables S1, S3). After analyses, one of the miRNAs, miR408, was noticed due to the wound-repressed expression. The abundance of miR408 decreased 32% in sweet potato after wounding (Table 1). In addition, its complementary miRNA strand, miR408*, predicted by Mfold (Zuker, 2003) was also found in the small RNA deep sequence libraries (Table 1). Most miRNA* is generally considered a by-product of the miRNA:miRNA* duplex and is typically degraded rapidly. The leader strand (miRNA) is generally highly expressed compared with the passenger strand (miRNA*) (Pérez-Quintero *et al.*, 2012). In this study, the miR408:miR408* ratio was ~100:1 based on the small RNA deep sequencing data. To isolate the miR408 precursor (pre408) in sweet potato, the sequence of miR408 was searched against a sweet potato transcriptomic database (Supplementary Table S4). A transcriptome contig perfectly matched with the sequence of miR408 was considered to be pre408. The secondary structure of pre408 was predicted by Mfold, and showed that the transcriptome contig formed a hairpin–loop structure with the mature miR408 in the stem region (Fig. 1A). These results strongly proved the presence of miR408 in sweet potato.

**Table 1.** Abundances and sequences of Ib-miR408-3p (Ib-miR408) and Ib-miR408* in sweet potato leaves

miRNA	**Sequence**	**Unwounded (RPM**)	**Wounded (RPM**)	**Ratio**
Ib-miR408	UGCACUGCCUCUUCCCUGGCU	290.95	197.04	0.68
Ib-miR408*	ACGGGGACGAGGCGGAGCAUG	2.7	3.77	1.4

RPM, reads per million.

Northern blot hybridization and small RNA qRT-PCR analyses were used to confirm the expression pattern of mature miR408. The result of northern blot indicated that the expression of miR408 was rapidly decreased by wounding, but was gradually restored to the basal level at 120 min of wounding in sweet potato leaves (Fig. 1B). Furthermore, the expression patterns of *pre408* and miR408 were verified by qRT-PCR (Fig. 1C, D), indicating that they were obviously decreased after wounding for 30–60 min. These findings demonstrated that the expression of *pre408* and miR408 was consistent with the small RNA sequencing result, within which miR408 expression was repressed by wounding.

Wounding in plants is mostly caused by insect herbivory. Thus, a comparison of miR408 expression between mechanical wounding and insect feeding was performed. The qRT-PCR analysis revealed that *pre408* and miR408 were down-regulated by *S. litura* feeding (Fig. 1C, D). MiR408 expression was reduced at 15 min after insect feeding, and was maintained at a low level until 60 min. The expression patterns of miR408 with mechanical wounding and insect feeding suggested that miR408 functioned in both responses.

Many miRNAs are evolutionarily conserved in plants (Zhang *et al.*, 2006). Sequences were compared using ClustalX2 to determine the correlation of miR408 and its precursor RNA (pre-miRNA) in different plant species. The *pre408* sequence (*Ib-MIR408*) of sweet potato was compared with those of other plants (Supplementary Fig. S1), indicating the diverse sequences of *pre408* observed among plants. Although miR408 was conserved in different plants, only nta-miR408, stu-miR408b-3p, cca-miR408, and ppt-miR408 were perfect matches with Ib-miR408. The sequences of other miR408s showed the two nucleotide differences at the 5' and 3' ends of miR408 (Supplementary Table S5). The nucleotide differences in mature miR408s may result in the recognition of various target genes in plants.

### Isolation and validation of miR408-targeting mRNAs in sweet potato

The identification of miRNA-targeting mRNAs is essential for the functional characterization of miRNAs (Karlova *et al.*, 2013). Plant miRNAs require strict sequence complementarity with their target genes to cleave at the pairing sites (Voinnet, 2009; Zhao *et al.*, 2015). The miR408 sequence was searched against the sweet potato transcriptome contig data set to predict potential miR408 targets. The penalty score was calculated from the number of mismatches (1 point) and GU wobbles (0.5 point) to predict the miRNA targets (Meyers *et al.*, 2008; Liu *et al.*, 2014). The candidate contigs by score (≤3 points) and no mismatch in the central region, the ninth, 10th, and 11th nucleotides from the 5' end of miRNA, were selected for further study. Based on these criteria, not only the typical miR408 target gene, *plantacyanin* (*IbPCL*), but also two sweet potato-specific genes, *3-ketoacyl-CoA synthase 4* (*IbKCS*) and *galacturonosyltransferase 7-like* (*IbGAUT*), were suggested to be the putative target genes of miR408 in sweet potato (Table 2). Hence, these three target genes, namely *IbKCS*, *IbPCL*, and *IbGAUT* , were selected, and their full lengths were obtained by RACE.

**Table 2.** Prediction of miR408 target genes from *de novo* transcriptome of sweet potato.

Annotation subject title	**Penalty Score**	**5' region**	**3' region**	**Central region**
*3-Ketoacyl-CoA synthase 4-like*	2	0/1^*a*^	0/1	0/0
*Plantacyanin-like (basic blue*)	2.5	0/0	1/2	0/0
*Galacturonosyltransferase 7-like*	3	1/1	1//1	0/0

^*a*^ The number of GU wobbles/the number of mismatches.

**Fig. 2.**
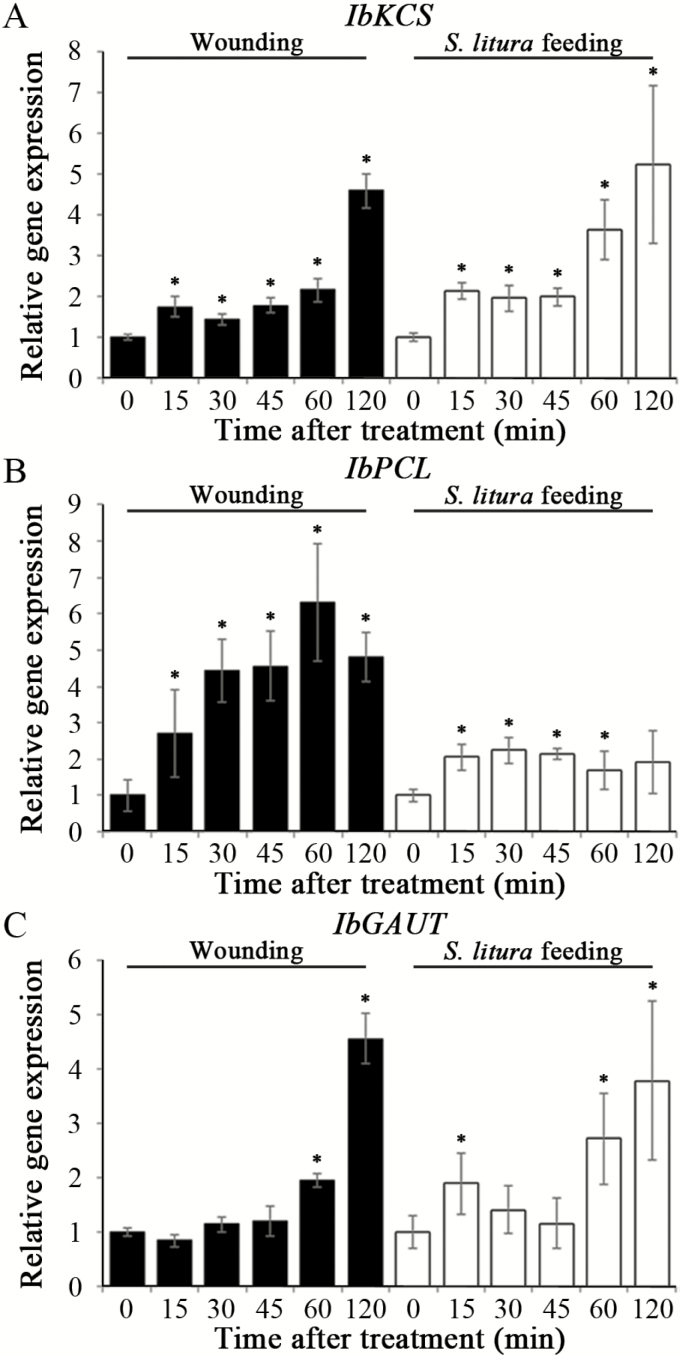
Expression patterns of miR408 target genes upon wounding and insect feeding. The expression of miR408 targets, *IbKCS* (A), *IbPCL* (B), and *IbGAUT* (C) in the leaves of sweet potato wounded by mechanical wounding or insect feeding for 0, 15, 30, 45, 60, and 120 min were analyzed by qRT-PCR. The qRT-PCR results were normalized by the expression levels of *IbActin*. Data are presented as means ±SD (*n*=4). The asterisks represent a significant difference from unwounded treatment by Student’s *t*-test (*P<0.05).

To determine the regulatory relationship between miR408 and its putative targets, the expression levels of *IbKCS*, *IbPCL*, and *IbGAUT* were monitored by qRT-PCR in sweet potato upon wounding. *IbKCS* and *IbPCL* expression was up-regulated rapidly, while *IbGAUT* expression was induced after wounding for 60 min (Fig. 2A–C). These results suggested that the expression patterns of miR408 putative targets were inversely related to that of miR408 in sweet potato upon mechanical wounding. To confirm further the expression of these putative targets in response to insect feeding, the expression levels of *IbKCS*, *IbPCL*, and *IbGAUT* were analyzed by qRT-PCR after insect feeding (Fig. 2A–C). The expression patterns of *IbKCS* and *IbGAUT* were similar between the mechanical wounding and insect feeding treatments, while the expression of *IbPCL* was slightly increased upon insect feeding. These results suggested that both mechanical wounding and herbivore wounding reduced the expression of miR408, resulting in the induction of *IbKCS*, *IbPCL*, and *IbGAUT* expression in sweet potato.

**Fig. 3.**
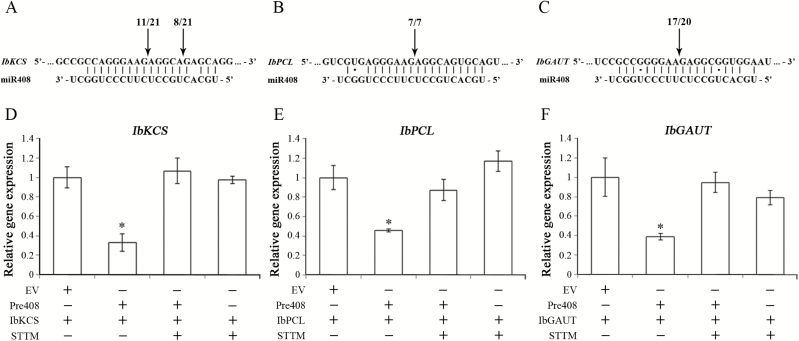
Validations of miR408 target genes by RACE and *Agrobacterium*-mediated transient assays. The potential cleavage sites of miR408 on three targets, *IbKCS* (A), *IbPCL* (B), and *IbGAUT* (C), in sweet potato were analyzed by 5'-RLM-RACE and 3'-PPM-RACE. The arrows indicate the positions of the cleavage sites, and clone frequencies are indicated by the numbers. In addition, *Agrobacterium*-mediated transient expression assays of miR408 and its targets, *IbKCS*, *IbPCL*, and *IbGAUT*, in tobacco leaves. Tobacco leaves were infiltrated with agrobacteria with a vector containing the miR408 targets, *IbKCS* (*35S:IbKCS*), *IbPCL* (*35S:IbPCL*), or *IbGAUT* (*35S:IbGAUT*), and with a vector containing the empty vector (EV), the miR408 precursor (Pre408), the short tandem target mimic of miR408 (STTM), or miR408 plus STTM. The expression of *IbKCS* (D), *IbPCL* (E), and *IbGAUT* (F) in tobacco leaves was then analyzed by qRT-PCR, and normalized to the levels of *NPTII* expression. The ratios relative to the plants with empty vector are shown as relative expression levels. Data are presented as means ±SD (*n*=4). The asterisks represent a significant difference by Student’s *t*-test (*P<0.05).

The interaction between miR408 and its targets were further examined. The 5'-RLM-RACE and 3'-PPM-RACE methods were used to identify the cleavage sites within the target mRNAs caused by miRNA (Lin *et al.*, 2012, 2013; C. Wang *et al.*, 2013). The RLM-RACE analyses revealed that *IbKCS*, *IbPCL*, and *IbGAUT* were mainly cleaved by miR408 at the 10th, 10th, and 11th nucleotides, respectively, relative to the 5' end of miR408 (Fig. 3A–C). These results demonstrated that *IbKCS*, *IbPCL*, and *IbGAUT* were miR408 target genes in sweet potato.

The *Agrobacterium*-mediated transient expression assay was performed to study the interaction between miR408 and its targets. The coding sequences of *IbKCS*, *IbPCL*, and *IbGAUT* were co-expressed with *pre408*, the short tandem target mimic of miR408 (STTM), or the empty vector (EV) in tobacco leaves by *Agrobacterium* infiltration. Expression of *IbKCS*, *IbPCL*, and *IbGAUT* was reduced by co-expression with *pre408* compared with those with the EV (Fig. 3D–F). The expression of *IbKCS*, *IbPCL*, and *IbGAUT* increased significantly in the presence of both *pre408* and STTM. These results provided further evidence that *IbKCS*, *IbPCL*, and *IbGAUT* were miR408 targets.

### Overexpression of miR408 reduces the resistance of plants to insect feeding

**Fig. 4.**
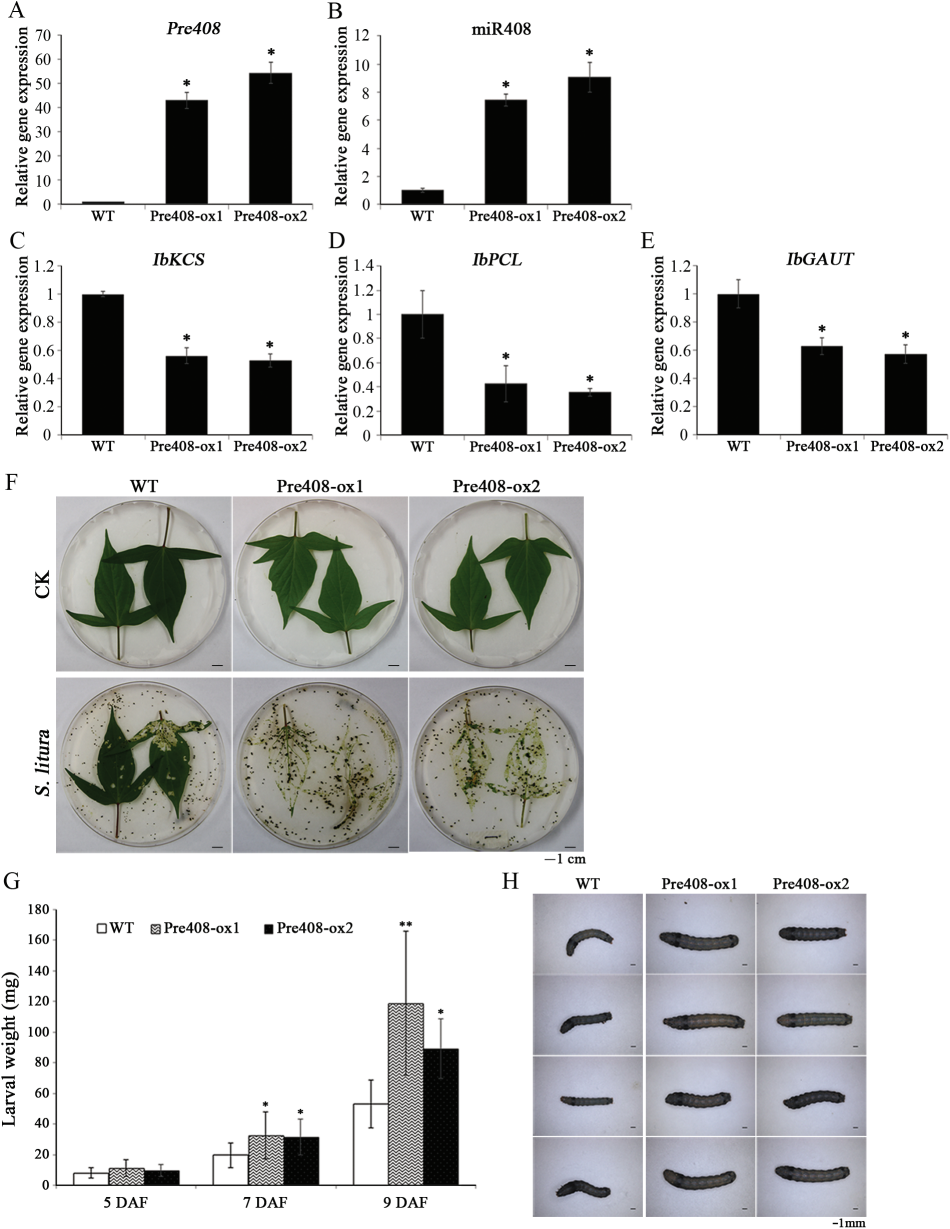
Gene expression and insect resistances of sweet potato overexpressing miR408. Expression levels of *pre408* (A), miR408 (B), *IbKCS* (C), *IbPCL* (D), and *IbGAUT* (E) in wild-type (WT) and miR408-overexpressing (Pre408-ox1 and Pre408-ox2) sweet potato were analyzed by qRT-PCR. Sweet potato leaves were wounded by tweezers for 120 mins. The expression level of 5.8S rRNA was used as an internal control for miR408 expression. *IbACT* expression levels were used as internal controls for *pre408* and miR408 target expression. Data are presented as means ±SD (*n*=4). The asterisks represent a significant difference by Student’s *t*-test (*P<0.05). (F) Representative leaves of WT, Pre408-ox1, and Pre408-ox2 sweet potato before (CK) and after feeding with *S. litura*. (G) Body weights of the second-instar larvae fed WT or transgenic plants were measured at 5, 7, and 9 d after feeding (DAF). Data are presented as means ±SD (*n*≥10). The asterisks represent a significant difference between WT and miR408-ox plants by Student’s *t*-test, *P<0.05; **P<0.01). (H) Representative images of the larvae fed WT or miR408-ox plants after 9 d.

To study the biological function of miR408 in sweet potato, transgenic sweet potato plants overexpressing miR408 driven by the 35S promoter were generated by *Agrobacterium*-mediated transformation. The expression levels of *pre408* and miR408 increased significantly in both transgenic plants (miR408-ox) compared with those of the wild type upon wounding (Fig. 4A, B). Thus, these two transgenic lines (Pre408-ox1 and Pre408-ox2) were selected to investigate the role of miR408 in detail. The expression of *IbKCS*, *IbPCL*, and *IbGAUT* was decreased in plants overexpressing miR408 (Fig. 4C–E), suggesting a negative correlation between miR408 and its target gene expression. The combination of transgenic studies and cleavage site identification clearly demonstrated that miR408 negatively regulates the expression of *IbKCS*, *IbPCL*, and *IbGAUT in vivo.*

Wounding stress is mainly caused by insect feeding in field crops. To study whether miR408 functions in defense against insect damage, the insect bioassay was performed. Second-instar larvae of *S. litura* were fed miR408-ox and mature wild-type sweet potato leaves. The leaves of plants overexpressing miR408 were consumed significantly more frequently by larvae than those of the wild type (Fig. 4F). The body weights of larvae fed the miR408-ox leaves were higher than those fed wild-type leaves at 7 d and 9 d of feeding (Fig. 4G, H), suggesting that miR408 attenuates plant resistance to insect attack.

**Fig. 5.**
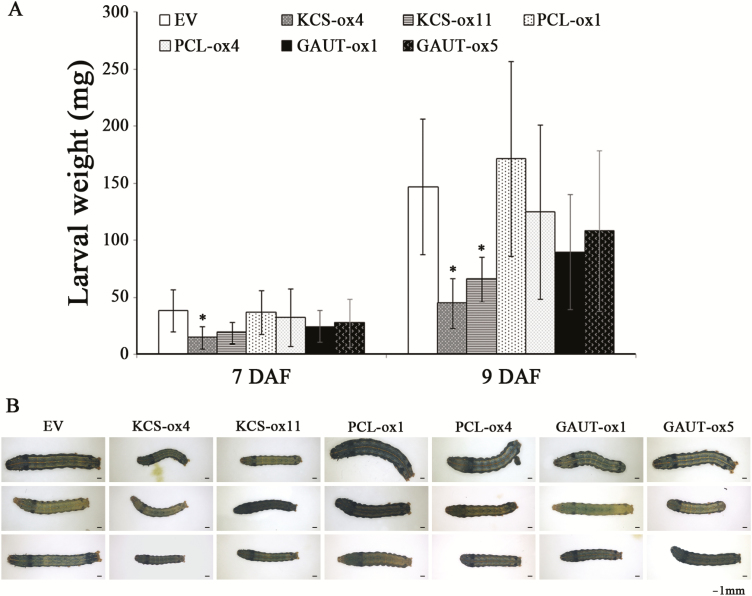
Insect resistance of *IbKCS*-overexpressing, *IbPCL*-overexpressing, and *IbGAUT*-overexpressing tobacco plants. (A) Body weights of the second-instar larvae were measured at the indicated time after larvae were fed the leaves from transgenic tobacco with empty vector (EV), and overexpressing *IbKCS* (KCS-ox4 and KCS-ox11), overexpressing *IbPCL* (PCL-ox1 and PCL-ox4), and overexpressing *IbGAUT* (GAUT-ox1 and GAUT-ox5). (B) Representative images of the larvae fed EV, KCS-ox, PCL-ox, and GAUT-ox tobacco leaves after 9 d. Data are presented as means ±SD (*n*≥8). The asterisks represent a significant difference between EV and overexpressing tobacco plants by Student’s *t*-test (*P<0.05).

To determine how miR408 influenced the plant defense against insect feeding, transgenic tobacco plants overexpressing miR408-targeting genes were generated. Transgenic tobacco plants overexpressing *IbKCS* (KCS-ox4 and KCS-ox11), *IbPCL* (PCL-ox1 and PCL-ox4), and *IbGAUT* (GAUT-ox1 and GAUT-ox5) were identified by RT-PCR and qRT-PCR (Supplementary Fig. S2), and their effects on insects were analyzed. The body weights of larvae fed IbKCS-ox leaves were lower than those fed EV leaves (Fig. 5A), whereas the body weights of insects fed IbPCL-ox and IbGAUT-ox leaves were not different from those fed the EV tobacco leaves (Fig. 5A). Moreover, *S. litura* larvae fed the EV tobacco leaves grew stronger than those fed the IbKCS-ox tobacco leaves (Fig. 5B), suggesting the important role of *IbKCS* in plant defense. These results implied that the insect defense related to miR408 may be co-ordinated by the function of *IbKCS*.

### Involvement of miR408 in JA response

**Fig. 6.**
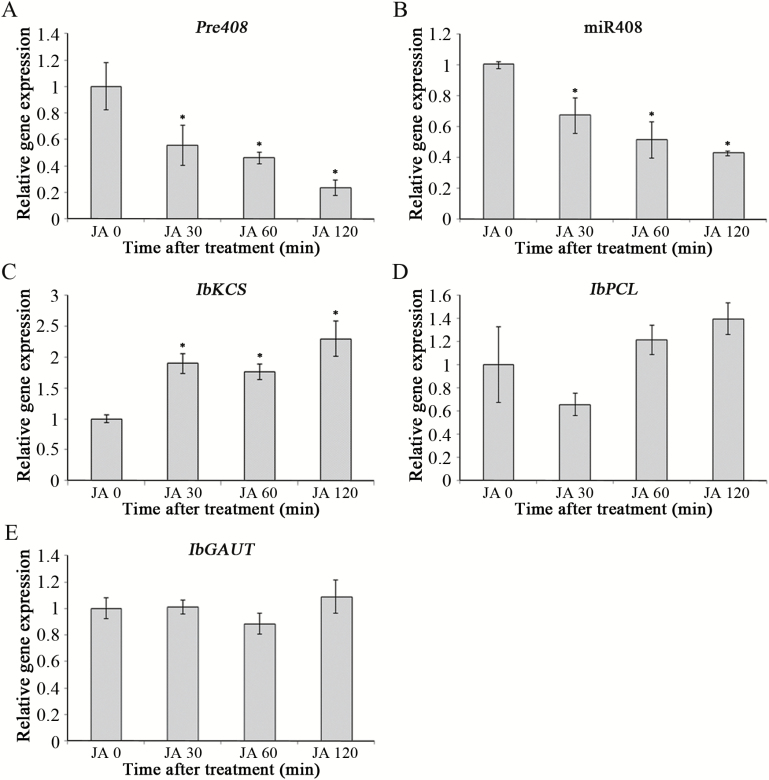
Expression patterns of *pre408*, miR408, and its targets after JA treatment. Total RNAs were extracted from sweet potato leaves sprayed with 50 µM MeJA for 0, 30, 60, and 120 min. The expression patterns of *pre408* (A), miR408 (B), *IbKCS* (C), *IbPCL* (D), and *IbGAUT* (E) were determined by qRT-PCR. The expression of 5.8S rRNA was used as an internal control for normalization of miR408 expression. The expression of *IbACT* was used as an internal control for normalization of *pre408* and its target genes. Data are indicated as means ±SD (*n*=4). The asterisks represent a significant difference by Student’s *t*-test (*P<0.05).

The defense-related phytohormone JA is known to participate in wounding signaling and insect damage of plants (Fürstenberg-Hägg *et al.*, 2013; Wasternack and Hause, 2013). To characterize whether miR408 is involved in JA signaling, the expression of miR408 and its target genes was examined after JA treatment (Fig. 6). The expression of *pre408* and miR408 was obviously reduced after JA treatment (Fig. 6A, B). Although *IbKCS*, *IbPCL*, and *IbGAUT* were the targets of miR408 in the wounding response, only *IbKCS* was regulated by JA treatment (Fig. 6C–E).

### Effects of miR408 on water loss rate, plant growth, and chlorophyll degradation

**Fig. 7.**
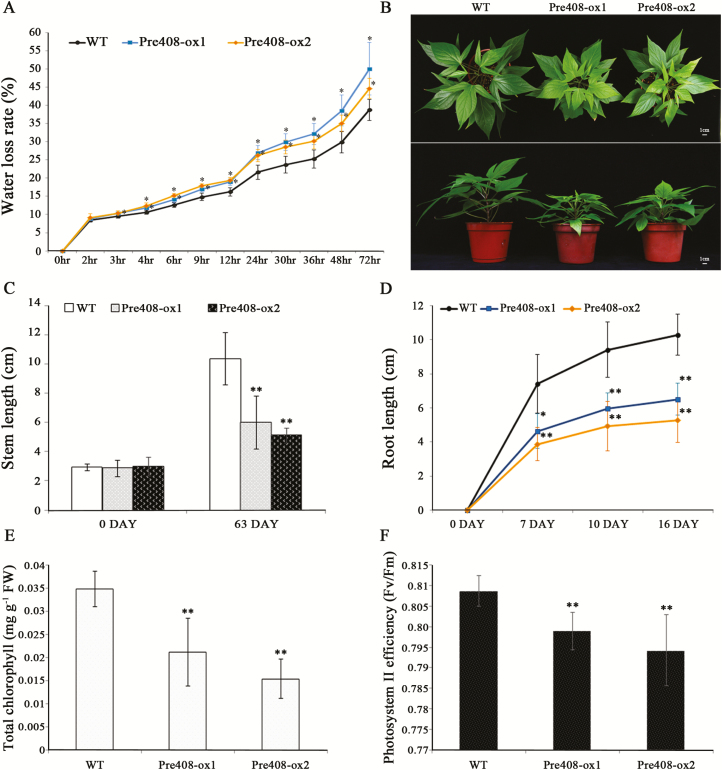
The phenotypes of miR408-overexpressing and wild-type (WT) sweet potato. (A) Water loss rates of WT and miR408-overexpressing (Pre408-ox1 and Pre408-ox2) plants. The water loss rates of the detached sweet potato leaves are shown. (B) The phenotypes of WT and miR408-overexpressing transgenic plants. Plants were grown for 6 weeks under a 16 h light/8 h dark photoperiod before photography. The stem lengths (C), root lengths (D), total chlorophyll contents (E), and PSII efﬁciencies (F) of WT and miR408-overexpressing sweet potato were measured. Data are presented as means ±SD (*n*≥6). The asterisk represents a significant difference between WT and miR408-ox plants by Student’s *t*-test (*P<0.05; **P<0.01).

Physical barriers function to protect plants against herbivory and water loss (Riederer and Schreiber, 2001; Lee *et al.*, 2009; Mitchell *et al.*, 2016). To determine whether miR408 is involved in barrier formation, the water loss rate of leaves was monitored. The water loss rate from the detached leaves of miR408-ox plants was increased compared with that of wild-type plants (Fig. 7A). This result indicated that overexpression of miR408 increased the sensitivity to water deﬁciency in sweet potato.

In addition to regulating defense ability and water deficiency, the transgenic lines Pre408-ox1 and Pre408-ox2 reduced vegetative growth (Fig. 7B). After 9 weeks growth of cutting propagation, stem lengths of miR408-ox transgenic sweet potatoes were ~40% shorter than those of the wild type (Fig. 7C). MiR408-ox also had a profound effect on root formation. Cut stems of plants with six fully developed leaves were immersed in water, and root growth was induced. After 16 d of inducing root growth, the length of the primary root of miR408-ox transgenic sweet potato was ~50% shorter than that of the wild type (Fig. 7D; Supplementary Fig. S3). These results indicated that overexpression of miR408 in sweet potato significantly decreased plant growth.

Furthermore, Pre408-ox1 and Pre408-ox2 plants had the visible chlorosis phenotypes; thus, the chlorophyll contents of the plants were analyzed. Total chlorophyll contents in miR408-ox plants were lower than those in the wild type (Fig. 7E). Simultaneously, the efﬁciency of PSII in transgenic sweet potato leaves was investigated by the *F*_v_/*F*_m_ values. Under a saturating light pulse, the *F*_v_/*F*_m_ value of miR408-ox was lower than that of the wild type, indicating the reduced efficiency of electron transfer in miR408-ox transgenic plants (Fig. 7F).

## Discussion

### MiR408 in plants

MiR408 contains 21 nucleotides and has been identiﬁed in >30 plants (Reinhart *et al.*, 2002; Axtell and Bowman, 2008; Kozomara and Griffiths-Jones, 2011). The alignment of pre408 sequences revealed the maximum conservation sequences in the stem region of the hairpin structures, from which mature miR408 are produced (Supplementary Fig. S1). Although miR408s within pre408s are conserved in many plants, the mature miR408 sequences are not all the same (Supplementary Table S5). The sequence of miR408 in sweet potato is exactly the same as that of nta-miR408, stu-miR408b-3p, cca-miR408, and ppt-miR408. MiRNA nucleotide variation originates either from different precursors transcribed from the *MIR* gene family (Jeong *et al.*, 2013; Jagtap and Shivaprasad, 2014) or from a single precursor by differential processing among plants (Starega-Roslan *et al.*, 2011; Jeong *et al.*, 2013). Moreover, the sequence of sweet potato miR408 has a two nucleotide difference from those of *Arabidopsis thaliana*, *Oryza sativa*, and *Triticum aestivum* (Supplementary Table S5). Recent studies have shown that miRNA families have a pattern of positional differentiation between dicots and monocots (Jagtap and Shivaprasad, 2014; Montes *et al.*, 2014). In general, the 5' start nucleotide of miR408 in most dicots is the nucleotide A, whereas that in monocots is the nucleotide C. However, the 5' start nucleotide and the 3'-terminal nucleotide of miR408 in sweet potato and *Solanaceae* are the nucleotide U (Supplementary Table S5). The shift variants of miRNA in sweet potato may lead to the recognition of different targets compared with other dicots.

The expression of miR408 in responses to cold, salinity, dehydration, and oxidative stresses, heavy metals, and pathogen infection has been reported in various plant species (Jia *et al.*, 2009*b*; Kantar *et al.*, 2010; Feng *et al.*, 2013; Ozhuner *et al.*, 2013; Jovanović *et al.*, 2014; Wu *et al.*, 2014; Ma *et al.*, 2015). However, research focusing on the role of miR408 in the wounding response is rare. MiR408 was identified as a wound-inducible miRNA in *Nicotiana attenuate* (Bozorov *et al.*, 2012), but its function in the wounding response remains unknown. Interestingly, the present study indicated that sweet potato miR408 was negatively regulated upon wounding, suggesting that although the miR408 sequence between *Solanaceae* and sweet potato is identical, the different expression patterns of miR408 among plants may indicate a special physiological role for miR408 in response to stress.

### Targets of miR408 in sweet potato

MiR408 target genes, including plantacyanin, uclacyanin, cupredoxin, laccase family genes, chemocyanin-like protein gene, and Timing of CAB expression 1, have been characterized in plants (Abdel-Ghany and Pilon, 2008; Trindade *et al.*, 2010; Feng *et al.*, 2013; Ma *et al.*, 2015; Thatcher *et al.*, 2015; Zhao *et al.*, 2016; Zhang *et al.*, 2017). In addition to the typical target *IbPCL*, two sweet potato-specific miR408 target genes, *IbKCS* and *IbGAUT*, were identified and verified in this study.

Sweet potato PCL exhibited 69% identity with the Arabidopsis PCL (basic blue protein) (Supplementary Figs S4, S5). MiR408 directly cleaved *IbPCL* at the 10th nucleotide from the 5' end of miR408 in sweet potato (Fig. 3B), and the same cleavage site caused by miR408 has been reported in others plants (Sunkar and Zhu, 2004; Maunoury and Vaucheret, 2011; Feng *et al.*, 2013; Mutum *et al.*, 2013; Hajyzadeh *et al.*, 2015). IbPCL contains the conserved domain of the type 1 copper-binding site of AtPCL (plantacyanin), AtUCC2 (uclacyanin II), and AtCPC (cupredoxin superfamily protein) (Supplementary Fig. S4). Plantacyanins belong to the phytocyanin family, which encodes blue copper proteins that function as electron transfer shuttles among proteins (Rydén and Hunt, 1993; Nersissian *et al.*, 1998; Ma *et al.*, 2015). In addition, plantacyanins are also stress-related proteins that function in copper starvation (Kim *et al.*, 2003), plant defense (Nersissian *et al.*, 1998), programmed cell death (Dong *et al.*, 2005), and heavy metal accumulation (Ruan *et al.*, 2011). These findings suggested that the expression of *IbPCL* may have an important role in wounding response in sweet potato.

In contrast to the interaction between *IbPCL* and miR408, *IbKCS* is an atypical target gene of miR408 that contains two mismatches within the miR408 sequence. The *IbKCS* cleavage sites directed by miR408 were located at the fifth and 10th nucleotides, which are non-canonical and canonical cutting sites, respectively, from the 5' end of miRNA (Fig. 3A). Generally, the extensive cleavage sites of target mRNA directed by miRNA are localized at the 10th and 11th nucleotides from the 5' end of miRNA (Schwab *et al.*, 2005; Liu *et al.*, 2015). However, non-canonical cleavage sites in targets induced by miRNA have been reported (Hackenberg *et al.*, 2015; Sharma *et al.*, 2016; Ferdous *et al.*, 2017; Shen *et al.*, 2017). To investigate the relationship between miR408 and *IbKCS* orthologous genes, the nucleotide sequences of *KCS* orthologs among plants were compared (Supplementary Fig. S6). The number of mismatches within the predicted complementary region of *KCS* orthologs in *N. tabacum*, *A. thaliana*, and *O. sativa* is more than five in the miR408 recognition region (Supplementary Fig. S6), indicating that regulation of miR408 in these *IbKCS* orthologos may not be effective.

*IbGAUT* is another atypical target of miR408 in sweet potato. MiR408 directed the cleavage of *IbGAUT* at the 11th nucleotide from the 5' end of miRNA (Fig. 3C). The nucleotide sequence comparison of *GAUT* orthologs revealed the presence of more than five mismatches, and the asymmetric bulges between miR408 and the miR408 predicted target sites in *N. tabacum*, *A. thaliana*, and *O. sativa* (Supplementary Fig. S7). These results suggested a unique feature of sweet potato miR408 by targeting to *IbKCS* and *IbGAUT* in regulation of the wounding response.

### Functions of sweet potato miR408 in the mechanical and herbivore wounding responses

In this study, we found that overexpression of miR408 in sweet potato decreased the resistance of sweet potato to *S. litura* (Fig. 4), indicating that the wound-induced repression of miR408 was a defense regulatory mechanism to prevent the wounding damage caused by insect feeding through miRNA-mediated target repression. JA plays an important role as a signaling molecule in plant defense against insect herbivory (Fürstenberg-Hägg *et al.*, 2013; Wasternack and Hause, 2013). Mithöfer *et al.* (2014) reported that both mechanical wounding and herbivore damage can increase JA accumulation in plants. Moreover, transcriptome analyses indicated that a large portion of wounding- and herbivore-induced responses are mediated through JA signaling (Reymond *et al.*, 2000; Wu and Baldwin, 2010).

Interestingly, a previous report showed that nta-miR408 is induced by wounding, but it is JA independent (Bozorov *et al.*, 2012). In contrast to nta-miR408, the expression of *pre408* and miR408 is JA dependent in sweet potato (Fig. 6A, B), suggesting that Ib-miR408 and nta-miR408 play different roles in the wound-mediated JA response. In sweet potato, endogenous JA enhanced by wounding modulates the signal transduction and triggers the activation of defense genes in response to herbivore wounding (Rajendran *et al.*, 2014; Chen *et al.*, 2016*b*). Both wounding and insect feeding reduced the expression of miR408, resulting in the induction of *IbKCS*, *IbPCL*, and *IbGAUT* in sweet potato. However, only *IbKCS* was clearly induced when the plants were treated with JA compared with *IbPCL* and *IbGAUT* (Fig. 6C–E), suggesting that *IbKCS* may be the major target gene of miR408 in the wound-mediated JA response. Indeed, *IbKCS*-ox plants demonstrated an enhanced defense response against *S. litura* compared with the EV, *IbPCL*-ox, and *IbGAUT*-ox plants (Fig. 5). Taken together, these results indicated that herbivore wounding regulates miR408 repression via JA signaling that increases insect resistance by regulating *IbKCS* expression. However, the detailed mechanism of *IbKCS* in insect resistance is not fully understood.

A phylogenetic analysis revealed that IbKCS is closely associated with AtKCS4, AtKCS9, AtKCS16, and AtKCS20 (Supplementary Fig. S8). These KCS proteins function as fatty acid elongases, which are involved in the synthesis of tetracosanoic acids as precursors of cuticular waxes, suberins, sphingolipids, and phospholipids (Kim *et al.*, 2013). In addition, KCS catalyzes acyl elongation to produce very long chain fatty acids for wax synthesis (Todd *et al.*, 1999; Weidenbach *et al.*, 2014; Guo and Jetter, 2017). In several cases, *KCS* genes are involved in the biosynthesis of cuticular wax, which covers the aerial surfaces of plants to limit water loss and invader attack (Kunst and Samuels, 2003; Jetter *et al.*, 2007; Lee *et al.*, 2009; War *et al.*, 2012; Weidenbach *et al.*, 2015). Plant barriers, including waxes, trichomes, and lattices, play important roles in defenses against phytophagous insects (Eigenbrode and Espelie, 1995; Reina-Pinto and Yephremov, 2009; Fürstenberg-Hägg *et al.*, 2013) and resistance to other stresses (Jenks *et al.*, 1995; De Bigault Du Granrut and Cacas, 2016). Previous literature demonstrated that the gene functioning in barrier trichome formation increases the ability for defense against insect herbivores in tobacco (Wang *et al.*, 2001) and tomato (Gao *et al.*, 2017). Although many studies discussed the involvement of the barrier of cuticular wax in plant resistance (Gorb and Gorb, 2017; Domínguez *et al.*, 2017), rare reports indicated that KCS genes or their homologs function directly in insect resistance. Thus, the detailed function of KCS in insect defense is still unclear. Moreover, the sensitivity to water loss in miR408-ox plants may also imply the function of IbKCS in wax synthesis (Fig. 7A). In view of these findings, we speculated that *IbKCS* may participate in surface barrier formation to confer resistance in sweet potato.

*IbPCL* and *IbGAUT* are wound-responsive genes, but JA independent (Fig. 6D, E). The JA-dependent wounding response plays an essential role in plant protection (Howe *et al.*, 1996; Halitschke and Baldwin, 2003; Howe, 2004), while JA-independent wound-induced genes may also participate in wound healing (de Bruxelles and Roberts, 2001; Sasaki *et al.*, 2002). PCL has been demonstrated to be a regulator of copper homeostasis in plants (Abdel-Ghany and Pilon, 2008; Ma *et al.*, 2015) in order to participate in the multiple abiotic stresses and resistance to stripe rust (Feng *et al.*, 2013; Ma *et al.*, 2015), but its detailed function remains unknown. PCL has been proposed to be involved in the oxidative burst, which may occur in pathogen infection and mechanical wounding (Low and Merida, 1996; Nersissian *et al.*, 1998; Dong *et al.*, 2005). Reactive oxygen species function as signaling molecules participating in regulating development and defense responses in plants (Apel and Hirt, 2004). Herbivore-induced damage requires repair of the cell wall to prevent pathogen entry (War *et al.*, 2012). Accordingly, most cell wall repair genes have been reported to be up-regulated upon wounding (Pandey *et al.*, 2017). GAUT family genes are critical proteins for the synthesis of homogalacturonan, which is the most abundant pectic polymer in the primary cell wall of plant (Wolf *et al.*, 2009; L. Wang *et al.*, 2013). The sequence alignments of IbGAUT exhibited high identity to the GAUT7 family (Supplementary Fig. S9), suggesting that IbGAUT may function in cell wall structure for wound healing (Bouton *et al.*, 2002; Lao *et al.*, 2003; Orfila *et al.*, 2005; Persson *et al.*, 2007). Thus, we proposed that *IbPCL* and *IbGAUT* may play different roles from *IbKCS* in sweet potato upon wounding.

The conserved miR408 family has diverse biological functions in multiple plant species. Our study reveals the involvement of miR408 in the wounding response by regulating its specific targets in sweet potato. The regulation mechanism between miR408 and its targets, *IbKCS*, *IbPCL*, and *IbGAUT*, not only affects plant development but also participates in the plant defense response.

## Supplementary data

Supplementary data are available at *JXB* online.

Table S1. The conserved miRNAs repressed in sweet potato upon wounding by small RNA sequencing.

Table S2. Primers used in this study.

Table S3. Small RNA deep sequencing of the unwounded and wounded sweet potato leaves.

Table S4. Statistics of the paired-end transcriptome sequencing data from sweet potato leaves.

Table S5. Mature miR408 sequences in different plants.

Fig. S1. Clusters of *miR408 precursors* (*MIR408*) in different plant species.

Fig. S2. Analysis of transgenic plants.

Fig. S3. Phenotypes of sweet potato plants overexpressing miR408.

Fig. S4. Comparisons of the plantacyanin family.

Fig. S5. Phylogenetic tree of the basic blue protein (BBP) family in plants.

Fig. S6. Comparisons of the 3-ketoacyl-CoA synthase-like gene (*KCS*) in plants.

Fig. S7. Comparisons of galacturonosyltransferase-like gene (*GAUT*) in plants.

Fig. S8. Phylogenetic tree of the KCS protein family in plants.

Fig. S9. Phylogenetic tree of the GAUT protein family in plants.

## Supplementary Material

Supplementary_Material
