# Supplementary material for: MicroR408 regulates defense response upon wounding in sweet potato
Source: J Exp Bot. 2018 Nov 5;70(2):469–83. doi: 10.1093/jxb/ery381 (PMC6322576; doi:10.1093/jxb/ery381)
Supplement: Supplementary_Material [file ery381_suppl_supplementary_material.pdf]

**Supplementary Tables**

**Table S1. The conserved miRNAs repressed in sweet potato upon wounding by small RNA sequencings.**

| miRNA | Unwound (RPM) | Wounded (RPM) | Ratio |
|---|---|---|---|
| gma-miR390e | 1.84 | 1.47 | 0.80 |
| aau-miR396 | 1217.67 | 967.44 | 0.79 |
| aly-miR167d-5p | 155.48 | 123.42 | 0.79 |
| bna-miR397a | 1.23 | 0.96 | 0.78 |
| csi-miR166d | 13.81 | 10.66 | 0.77 |
| ata-miR171a-3p | 24.79 | 18.84 | 0.76 |
| gma-miR396h | 5.64 | 4.28 | 0.76 |
| gma-miR168b | 1.41 | 1.02 | 0.72 |
| aly-miR166a-3p | 61865.34 | 44743.07 | 0.72 |
| gma-miR156q | 19.51 | 14.11 | 0.72 |
| ahy-miR167-5p | 1.60 | 1.15 | 0.72 |
| aly-miR396b-5p | 1009.86 | 722.96 | 0.72 |
| aau-miR162 | 166.52 | 117.61 | 0.71 |
| atr-miR169a | 45.77 | 32.24 | 0.70 |
| stu-miR398a-3p | 33.25 | 23.37 | 0.70 |
| bdi-miR166e-3p | 208.92 | 146.66 | 0.70 |
| atr-miR398 | 39.33 | 27.52 | 0.70 |
| aqc-miR166a | 5423.01 | 3763.45 | 0.69 |
| aly-miR171a-5p | 1.84 | 1.28 | 0.69 |
| csi-miR166a | 13.68 | 9.45 | 0.69 |
| cca-miR408 | 290.95 | 197.04 | 0.68 |
| bdi-miR159b-3p.1 | 167.38 | 112.69 | 0.67 |
| aly-miR167b-3p | 11.78 | 7.92 | 0.67 |
| osa-miR166i-3p | 1.17 | 0.77 | 0.66 |
| cpa-miR166e | 33.75 | 22.16 | 0.66 |
| gma-miR403a | 8.41 | 5.43 | 0.65 |
| ata-miR398f-3p | 127.93 | 81.73 | 0.64 |
| mes-miR166i | 74.00 | 46.93 | 0.63 |
| gma-miR166m | 16.87 | 10.66 | 0.63 |
| bna-miR166f | 23.74 | 14.81 | 0.62 |
| aly-miR164a-5p | 8.53 | 5.04 | 0.59 |
| bdi-miR162 | 1.47 | 0.83 | 0.56 |
| aly-miR395d-3p | 0.92 | 0.51 | 0.56 |
| cme-miR396e | 9.57 | 5.24 | 0.55 |
| aly-miR165a-3p | 0.61 | 0.32 | 0.52 |

| miRNA | Unwound (RPM) | Wounded (RPM) | Ratio |
|---|---|---|---|
| zma-miR159h-3p | 0.86 | 0.45 | 0.52 |
| mtr-miR166b | 1.96 | 1.02 | 0.52 |
| bdi-miR530a | 3.80 | 1.92 | 0.50 |
| mdm-miR396a | 10.19 | 5.04 | 0.50 |
| aly-miR171b-3p | 1.17 | 0.57 | 0.49 |
| aqc-miR171a | 5.46 | 2.68 | 0.49 |
| ahy-miR156a | 0.67 | 0.32 | 0.47 |
| gma-miR396e | 1.35 | 0.57 | 0.43 |
| smo-miR408 | 1.53 | 0.64 | 0.42 |
| gma-miR390b-5p | 1.29 | 0.51 | 0.40 |
| cme-miR319c | 1.29 | 0.51 | 0.40 |
| pab-miR396b | 7.67 | 2.75 | 0.36 |
| ppt-miR166j | 1.10 | 0.38 | 0.35 |
| ppe-miR396a | 5.40 | 1.85 | 0.34 |
| pab-miR397 | 1.53 | 0.51 | 0.33 |
| tcc-miR399a | 6.20 | 1.66 | 0.27 |

*RPM (reads per million)

**Table S2. Primers used in this study.**

**Northern**

| | |
|---|---|
| T3 top strand | ATGAATTAACCCTCACTAAAG |
| T3-anti-miR408 | TGCACTGCCTCTTCCCTGGCTCTTTAGTGAGGGTTAATTCAT |
| 5.8S rRNA F | AAATACGACTCTCGGCAACGG |
| 5.8S rRNA R | AACTTGCGTTCAAAGACTCGAT |

**RT**

| | |
|---|---|
| MatureRT-1 | CGACTGGAGCACGAGGACACTGACATGGACTGAAGGAGTTTTTTTTTTTTTTTTTTVN |
| T25VN | TTTTTTTTTTTTTTTTTTTTTTTTTVN |

**RACE**

| | |
|---|---|
| 5-RNA adapter | rUrCrArGrUrGrUrArCrGrGrArArUrUrCrCrArUrG |
| 5-adapter | CAGTCAGTGTACGGAATTCCATG |
| 3-RT adapter | AGTCGGGATCCCGTTGACTTTTTTTTTTTTTTTTTTVN |
| 3-adapter | AGTCGGGATCCCGTTGAC |
| UPM long | CTAATACGACTCACTATAGGGCAAGCAGTGGTATCAACGCAGAGT |
| UPM short | CTAATACGACTCACTATAGGGC |
| IbKCS RACE-1 | CCTGTGCACTTGCAACAAATCCTTGGCT |
| IbKCS RACE-2 | GCCTATATCTTTAGGCTTCAAAGACG |
| IbKCS RLM | GTTATCGCCCTCCTGATAACCTC |
| IbPCL RACE | TCAGACCGGGAAAGACCGG |
| IbPCL RLM | CAGCCGCTGACGTTAAAGGTCC |
| IbGAUT RACE-1 | GACGCCATAAGCAGTAGCTCCC |
| IbGAUT RACE-2 | CGGCTGGATTCCAGGTGACGTGATC |
| IbGAUT RLM | CCTTTGGAAAAGTAGGTTCCAATCTTC |

**Quantitative RT-PCR primers**

| | |
|---|---|
| IbKCS F | GCGTAAGATCTTAGAGCGGTCTGGC |
| IbKCS R | GGAGTCGGATTGAAGAGACTACAATTCAC |
| IbPCL F | ATGATGTGCCGTCGTGAGGG |
| IbPCL R | GGTAGCTGGGCCCGTAGTTGAAAG |
| IbGAUT F | ATGAAGGGCGGTGGAGCGCCTTAC |
| IbGAUT R | GCTTCAGGTTGATCATAAACATGAACC |
| Pre408 F | AAGTTGGGAAGGCGGTGGA |
| Pre408 R | GAAAAAGAATGGAGGAAAGGAGAGCC |
| miR408 F | TGCACTGCCTCTTCCCTGGCTAAA |
| UniPCR 1 | CGACTGGAGCACGAGGACACTGA |
| IbActin F | GACTACCATGTTCCCCGGTA |
| IbActin R | TTGTATGCCACGAGCATCTT |
| NPTII F | GCGATGCCTGCTTGCCGAATA |
| NPTII R | ACCGTAAAGCACGAGGAAGCG |
| NtActin F | ACCTTGCTGGACGTGACCTTACTGAT |
| NtActin R | GTTGTCTCGTGGATTCCAGCAGCTT |

**Construction primers**

| | |
|---|---|
| Pre408 XbaI F | TCTAGAAAGTTGGGAAGGCGGTG |
| Pre408 SmaI R | CCCGGGGAGAAGAAAAAGAATGGAGGAAA |
| IbKCS XbaI F | TCTAGAATGGACGGCGGCGCGGAAGTCAAC |
| IbKCS XmaI R | CCCGGGCTATGACACTATTTTTACTGGATACCTGTC |
| IbPCL XbaI F | TCTAGACTCTTAGCTCTCAAGATGATGTGCCG |
| IbPCL SmaI R | CCCGGGGTCAAACAGCCGAGACAGCGATC |
| IbGAUT BamHI F | GGATCCATGAAGGGCGGTGGAGCGC |
| IbGAUT XmaI R | CCCGGGCTAATTCACATTGCAATCTCTTAAC |
| STTM-Mimic408-1 | TCTAGAAGCCAGGGAAGCTAAGGCAGTGCAGTTGTTGTTGTTATGGTCTAATTTAAAT |
| STTM-Mimic408-2 | ATTTAAATATGGTCTAAAGAAGAAGAATAGCCAGGGAAGCTAAGGCAGTGCACCCGGG |
| XbaI-STTM-Mimic408 1F | TCTAGAAGCCAGGGAAGCTAAG |
| XmaI-STTM-Mimic408 2R | CCCGGGTGCACTGCCTTAG |
| STTM-Mimic 1R | ATTTAAATTAGACCATAACAACAACAAC |
| STTM-Mimic 2F | ATTTAAATATGGTCTAAAGAAGAAGAAT |

**Table S3. Small RNA deep sequencings of the unwounded and wounded sweet potato leaves.**

| | Genome Analyzer IIx | |
|---|---|---|
| | W- | W+30 |
| **Before adapter trimming** | | |
| **Total reads** | 16538421 | 15987130 |
| **GC percentage (%)** | 51 | 52 |
| **Reads length** | 51 | 51 |
| **After adapter trimming** | | |
| **Total reads** | 16298335 | 15661977 |
| **GC percentage (%)** | 48 | 49 |
| **Reads length** | 1-51 | 1-51 |
| **Number of read count >1** | 259299 | 234732 |
| **Poly A, T, C, G, N** | 1053 | 752 |
| **Unique reads** | 258246 | 233980 |
| **Rfame matching** | 503 | 466 |
| **Known miRNA matching** | 91 | 90 |
| **Unmatched** | 222606 | 201846 |

**Table S4. Statistics of the paired-end transcriptome sequencing data from sweet potato leaves.**

| | Transcriptome | |
|---|---|---|
| | **Read 1** | **Read 2** |
| **Before adapter trimming** | | |
| **Total reads** | 21649749 | 21649749 |
| **GC percentage (%)** | 47 | 47 |
| **Reads length** | 251 | 251 |
| **After adapter trimming** | | |
| **Total reads** | 21644025 | 21648335 |
| **GC percentage (%)** | 48 | 48 |
| **Reads length** | 1-251 | 1-251 |
| **Unique reads** | 19438419 | 19903499 |
| **Mapping with contig** | | |
| **Mapping reads** | 16394651 | 15247754 |
| **Mapping rate** | 75.75% | 70.43% |

**Table S5. Mature miR408 sequences in different plants.**

| ID | Mature sequence | Species |
|---|---|---|
| **Ib-miR408** | UGCACUGCCUCUUCCCUGGCU | *Ipomoea batatas* |
| **nta-miR408** | UGCACUGCCUCUUCCCUGGCU | *Nicotiana tabacum* |
| **stu-miR408b-3p** | UGCACUGCCUCUUCCCUGGCU | *Solanum tuberosum L.* |
| **cca-miR408** | UGCACUGCCUCUUCCCUGGCU | *Cynara cardunculus* |
| **ppt-miR408** | UGCACUGCCUCUUCCCUGGCU | *Physcomitrella patens* |
| **ath-miR408-3p** | AUGCACUGCCUCUUCCCUGGC | *Arabidopsis thaliana* |
| **aly-miR408-3p** | AUGCACUGCCUCUUCCCUGGC | *Arabidopsis lyrata* |
| **mtr-miR408-3p** | AUGCACUGCCUCUUCCCUGGC | *Medicago truncatula* |
| **gma-miR408b-3p** | AUGCACUGCCUCUUCCCUGGC | *Glycine max* |
| **ahy-miR408-3p** | AUGCACUGCCUCUUCCCUGGC | *Arachis hypogaea L.* |
| **car-miR408** | AUGCACUGCCUCUUCCCUGGC | *Cicer arietinum L.* |
| **ptr-miR408** | AUGCACUGCCUCUUCCCUGGC | *Populus trichocarpa* |
| **osa-miR408-3p** | CUGCACUGCCUCUUCCCUGGC | *Oryza sativa* |
| **tae-miR408** | CUGCACUGCCUCUUCCCUGGC | *Triticum aestivum* |
| **zma-miR408** | CUGCACUGCCUCUUCCCUGGC | *Zea mays* |

## Supplementary Figures

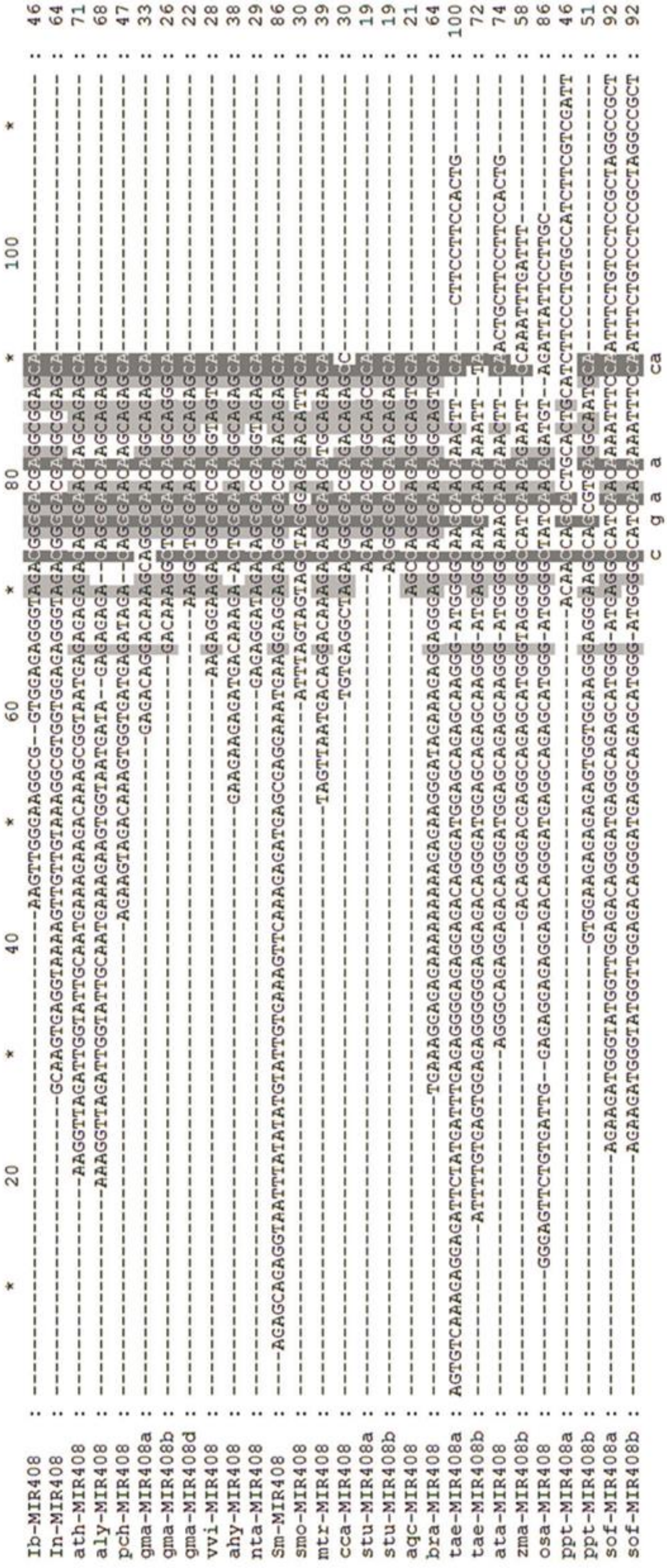

```
                        120       *       140       *       160       *       180       *       200       *       220
Ib-MIR408   : ---------TGAGATCGGCAGTT--------GGGGTTA----------------------------------AGTTGCTGCCCTTTCCA-TGCACTGCCTCTTCCCTGGCTCTC : 108
In-MIR408   : ---------TGAGATCGGCACTTCAACTTTCGGGGTTA----------------------------------AGTTGTTGTCCTTTCCA-TGCACTGCCTCTTCCCTGGCTCTC : 134
ath-MIR408  : ---------TGGATTCAGTTTACTAAAACATTAAACGAC--------------------------------TCTGTTTGTCTCTACCCA-TGCACTGCCTCTTCCCTGGCTCCC : 144
aly-MIR408  : ---------TGGATTCAGTTTACTAAAACATTAAACGAC--------------------------------TCTGTTTGTCTCTACCCA-TGCACTGCCTCTTCCCTGGCTCCC : 141
pch-MIR408  : ---------TGGATTCAGTTTACTAAAACATTAAACGAC--------------------------------TGTGTTTGTCTCTACCCA-TGCACTGCCTCTTCCCTGGCTCCC : 120
gma-MIR408a : ---------TGGATGCAGCTATCAACACAATATTGTCAAG-------AAACTGAGAGTGAGAG-GAGAAATATGTTGTGGTTCTGCTCA-TGCACTGCCTCTTCCCTGGCTCTG : 129
gma-MIR408b : ---------CGAATGCAGCTATCAACAGAAAATGGTAAAGTGAGAATGAAAGGAGAGAGAGAGAGAGAGATCTGTTGTGGCTACGCTCA-TGCACTGCCTCTTCCCTGGCTCTG : 130
gma-MIR408d : ---------CGAATGCAGCTATCAACAGAAAATGGAGAAGTGAGAA-ATGAGAGGAGAGAAAGAGAGAGATCTGTTAGGGCTACGCTCA-TGCACTGCCTCTTCCCTGGCTCTG : 125
vvi-MIR408  : ---------TGGATGCAACTATTAACAGAAGAATGTTAAG--------------------------------CTGTTTTGCTCTACCCA-TGCACTGCCTCTTCCCTGGCTCTG : 101
ahy-MIR408  : ---------TGAATGCAACT------ATCAATAGACACA-------------------------------TTTTGTTCATTGACGCTCA-TGCACTGCCTCTTCCCTGGCTCTC : 105
nta-MIR408  : ---------TGAGATCTGCAATT-------------------------------------------------TTTGCTTGCCCATTCTA-TGCACTGCCTCTTCCCTGGCTCTC :  84
Sm-MIR408   : ---------TGGGATCGACCTTTCAACACCATACATGG----------------------------------TTTTGTTGCCCTTTCCA-TGCACTGCCTCTTCCCTGGCTCCT : 156
smo-MIR408  : ---------TGATAGCTACACCAAAGAGCAACCTCGGCTC----------------------------------CTTGTGTGGCTTTGA-TGCACTGCCTCTTCCCTGGCTGCG : 100
mtr-MIR408  : ---------TGGATGAAACTATCGACAGTGAA--GGAAA--------------------------------CTGTTGTGGCTCCACTCA-TGCACTGCCTCTTCCCTGGCTTAT : 109
cca-MIR408  : ---------CGAAAAAGCCTAGTCAATACTTGGGTATG----------------------------------TATTGTTATCTTTTTCA-TGCACTGCCTCTTCCCTGGCTATC : 100
stu-MIR408a : ---------TGAGATCTACAATTTTAC-----------------------------------------------TAGTTGCCCATTCCA-TGCACAGCCTCTTCCCTGGTT--- :  73
stu-MIR408b : ---------TGAGATCTGCAATT-------------------------------------------------TTTACTTGCCTATTCCA-TGCACTGCCTCTTCCCTGGCT--- :  71
aqc-MIR408  : ---------TGGAAACAG----CAACAACAAT-ATTTG------------------------------TGTTGATTGG--TTCATTCCA-TGCTCTGCCTCATCCTTGTCTGT- :  87
bra-MIR408  : ---------TGGGAACAGAC--CAAACATAGTCATTTGAT---------------------------GTTTTGGTAAAAACTCAATCCA-TGCTCTGCTTGTTCCCTGTCTCTC : 139
tae-MIR408a : -----ATCACCACCTCATTAT--GACAAGA-GCGAGAGAGTTGTGAGAGATGAGAGGTTCCG-----ACTGTTGTTGTTGCTCCCTCCC-TGCACTGCCTCTTCCCTGGCTCCC : 200
tae-MIR408b : ---------CCACCTCATTAT--GAGAAGA-GGGAGAGAGTTGCCAGAG-------CTTCTG------TTGCTGTTGTTGCTCCCTCCC-TGCACTGCCTCTTCCCTGGCTCCC : 160
ata-MIR408  : -----ATCACCTCCTCATTATATGACAAGA-GCGAGAGAGTTGTGAGAGATGAGAGCTTTTGTTATTGTTGTTGTTGTTGCTCCCTCCC-TGCACTGCCTCTTCCCTGCCTCCC : 181
zma-MIR408b : ---------CTGTTTCCTCGC-TCACAAAATG-GAGGGACTCACCACAAACA----CACTCA--------GGCGTTGTTGCTCCCTCCCCTGCACTGCCTCTTCCCTGGCTC-- : 147
osa-MIR408  : ---------ACAAGACATGAT--GATGAGCTGTGAATGAGTTCTGAGAGATGG------CTG------GTGTTGTTGTTGCTCCCTCCCCTGCACTGCCTCTTCCCTGGCTCCC : 177
ppt-MIR408a : GTCTCGTACCGAAAGTGATTCAACCACAGCGAGATCAGACTCA---------------------------ACTGTTAAAGCTGATGACACTGATACGAGATGATGAAGACATAG : 133
ppt-MIR408b : ---------TGACAACAGCATGCCCAGGAGGTCCTGAGG--------------------------------------GTGTTGTCCTCA-TGCACTGCCTCTTCCCTGGCTTC- : 116
sof-MIR408a : ACTGCATTTCTGTTTCCTTGC-TCACAAAACG-GAGGGATTTGTGAGAGTTAT---CAGGCAGAAAGAACAAAGAAGGTGCCTCCCTGG-TGAAGTGGTGATGGCCTGACCTGA : 200
sof-MIR408b : ACTGCATTTATGTTTCCTTGC-TCACAAAACG-GAGGGATTTGTGAGAGTTAT---CAGGCAGAAAGAACAAAGAAGGTGCCTCCCTGG-TGAAGTGGTGATGGCCTGACCTGA : 200
                            g                                                           t            c  TGcactGcctcttccctGgct
```

```
                    *        240         *        260         *        280         *        300         *
Ib-MIR408    : CTTTCCTCCATTCTTTTTCTTCT--------------------------------------------------------------- : 131
In-MIR408    : CTTTCCTCCATTATTTTTCCTCTCTATGTTCATT---------------------------------------------------- : 168
ath-MIR408   : TCTTTT-TTTCTCTATATTTCTCTCTCTCCTTTCATTTCACAGCTTTCAATGGAATTTTATTGCTACTGCTAACG----------- : 218
aly-MIR408   : TCTTTTCTCTCTCTCTCTCTCTCTCCCTCTCTCTATTTCTTTCTCTCCTTTCATTTCAATGGATTTTT---------------- : 211
pch-MIR408   : TCTTTC-TCTCTAT-----TCTTTCTCTCCTTTT---------------------------------------------------- : 148
gma-MIR408a  : TCTC---------------------------------------------------------------------------------- : 133
gma-MIR408b  : TC------------------------------------------------------------------------------------ : 132
gma-MIR408d  : TCTCT--------------------------------------------------------------------------------- : 130
vvi-MIR408   : TCTCTC-------------------------------------------------------------------------------- : 107
ahy-MIR408   : TCTTTCTTTTTCCT-----TCT---------------------------------------------------------------- : 122
nta-MIR408   : CTCCCTC------------------------------------------------------------------------------- :  91
Sm-MIR408    : CTCTCTCTCTCTCTCTCAATTTCATTTCTATGTTGCTGGTGTGAAAGCCATATCATGTACACCAAAAAATTACTGATGCTTT---- : 238
smo-MIR408   : GCCAAGTTA----------------------------------------------------------------------------- : 109
mtr-MIR408   : AATCTGTTTTTATTAT---------------------------------------------------------------------- : 125
cca-MIR408   : ATCTTTC------------------------------------------------------------------------------- : 107
stu-MIR408a  : -------------------------------------------------------------------------------------- :   -
stu-MIR408b  : -------------------------------------------------------------------------------------- :   -
aqc-MIR408   : -------------------------------------------------------------------------------------- :   -
bra-MIR408   : TCTGATTACCACTTTTGTCTTCTTCATTT--------------------------------------------------------- : 168
tae-MIR408a  : CTCCCCTCCCATCTATCTCCCTCTTCCTCTCTTGCAGTCTTGCTATTTTCTGTCCCTGTTATCG---------------------- : 264
tae-MIR408b  : CTCCCAAATCTCTCCCTCCCCCTCTCT----------------------------------------------------------- : 187
ata-MIR408   : CTCCCA-------------------------------------------------------------------------------- : 187
zma-MIR408b  : -------------------------------------------------------------------------------------- :   -
osa-MIR408   : CTGCACACCTCTCTCTCTCTCTCTCTCTCTCTGTG--------------------------------------------------- : 212
ppt-MIR408a  : GCAGGGCAGCCAGTG----------------------------------------------------------------------- : 148
ppt-MIR408b  : -CCTACATAGCTCGCCATTCTTGTGCTCT--------------------------------------------------------- : 144
sof-MIR408a  : GACGGCTGAGAGCTCAGCTGGTGTCCTG---TTGTTGCTTCCTCCCCTGCACTGCCTCTTCCCTGGCTCCCCACCGTTGCCCTTGC : 283
sof-MIR408b  : GAGGGCTGAGAGCTCAGCTGGTGTCCTGGTGTTGTTGCTTCCTCCCCTGCACTGCCTCTTCCCTGGCTCCCCACCGTTGCCCTTGC : 286
```

**Fig. S1. Clusters of *miR408 precursors* (*MIR408*) in different plant species.**

The comparisons of *miR408 precursor* sequences among *Ipomoea batatas* (*Ib-MIR408*), *Ipomoea nil* (*In-MIR408*), *Arabidopsis thaliana* (*ath-MIR408*), *Arabidopsis lyrata* (*aly-MIR408*), *Pachycladon cheesemanii* (*pch-MIR408*), *Glycine max* (*gma-MIR408*), *Vitis vinifera* (*vvi-MIR408*), *Arachis hypogaea* (*ahy-MIR408*), *Nicotiana tabacum* (*nta-MIR408*), *Salvia miltiorrhiza* (*Sm-MIR408*), *Selaginella moellendorffii* (*smo-MIR408*), *Medicago truncatula* (*mtr-MIR408*), *Cynara cardunculus* (*cca-MIR408*), *Solanum tuberosum* (*stu-MIR408*), *Aquilegia caerulea* (*aqc-MIR408*), *Brassica rapa* (*bra-MIR408*), *Triticum aestivum* (*tae-MIR408*), *Aegilops tauschii* (*ata-MIR408*), *Zea mays* (*zma-MIR408*), *Oryza sativa* (*osa-MIR408*), *Physcomitrella patens* (*ppt-MIR408*) and *Saccharum officinarum* (*sof-MIR408*). Identical nucleotides are shaded by black color, and similar nucleotides are shaded by gray.

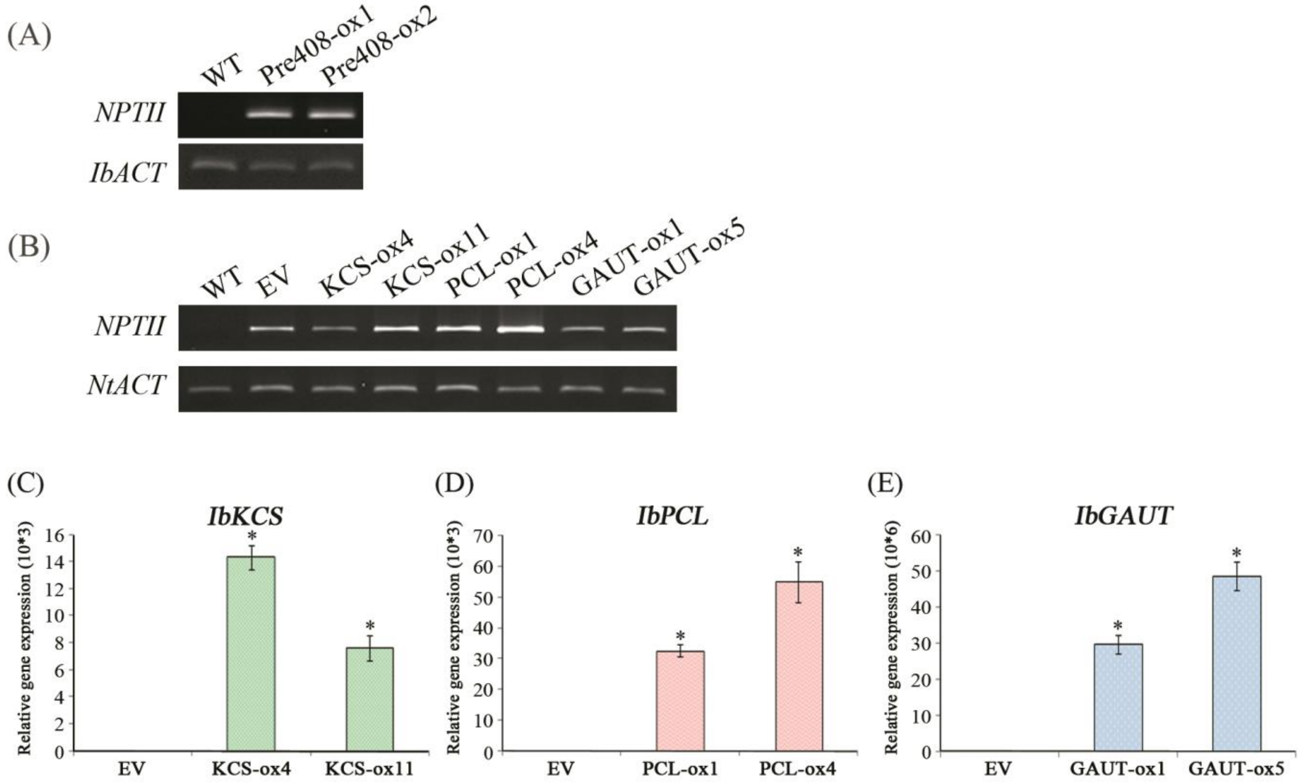


**Fig. S2. Analysis of transgenic plants.** RT-PCR analysis of transgenic sweet potato (A) and tobaccos (B) plants for *NPTII* gene and actin (control). Further, the expression level of *IbKCS* (C), *IbPCL* (D) and *IbGAUT* (E) in EV and *KCS*-ox, *PCL*-ox and *GAUT*-ox tobacco were determined by qRT-PCR. The expression of *NtACT* was used as an internal control. Data are indicated as means ± SD (n = 4). The asterisks represent the significant difference by Student's *t*-test (*P < 0.05).

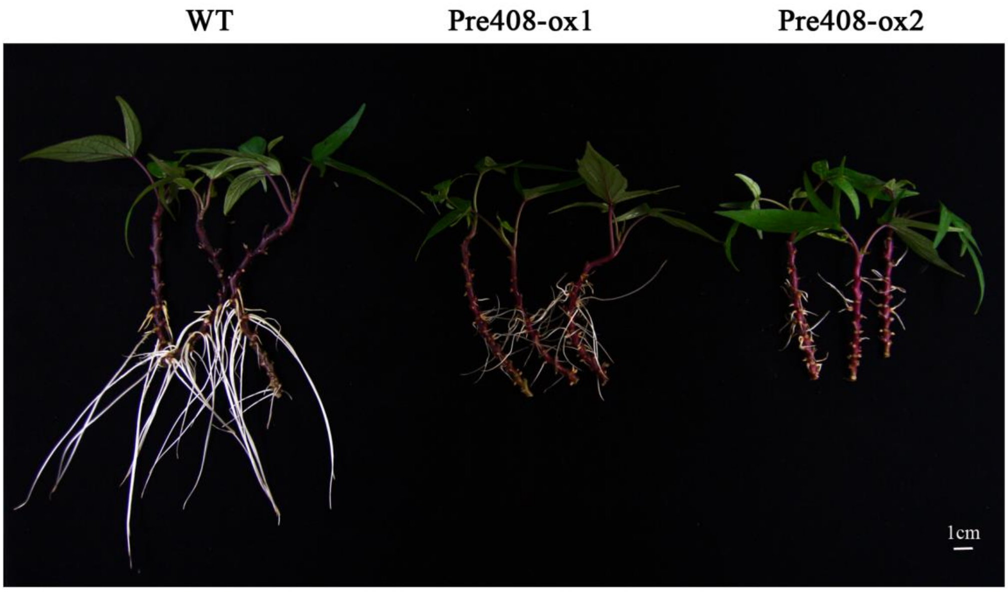


**Fig. S3. Phenotypes of sweet potato plants overexpressing miR408.** The root lengths of WT and miR408-ox plants were measured after the cuttage propagation of plants in water was performed for 7 d.

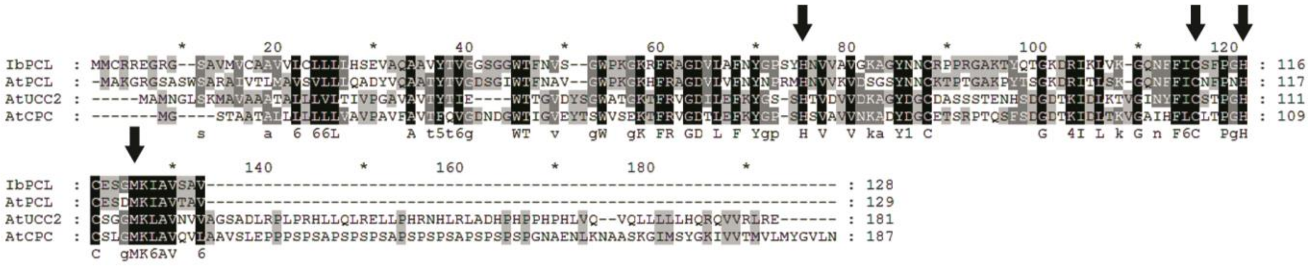

**Fig. S4. Comparisons of plantacyanin family.**

The comparisons of plantacyanin proteins between *Ipomoea batatas* (Ib) and *Arabidopsis thaliana* (At) are shown. The identical amino acid residues are shaded by black color, and the similar amino acid residues are shaded by gray. Downward arrows indicate the conserved domain of type 1 copper binding site of AtPCL (plantacyanin), AtUCC2 (uclacyanin II) and AtCPC (cupredoxin superfamily protein).

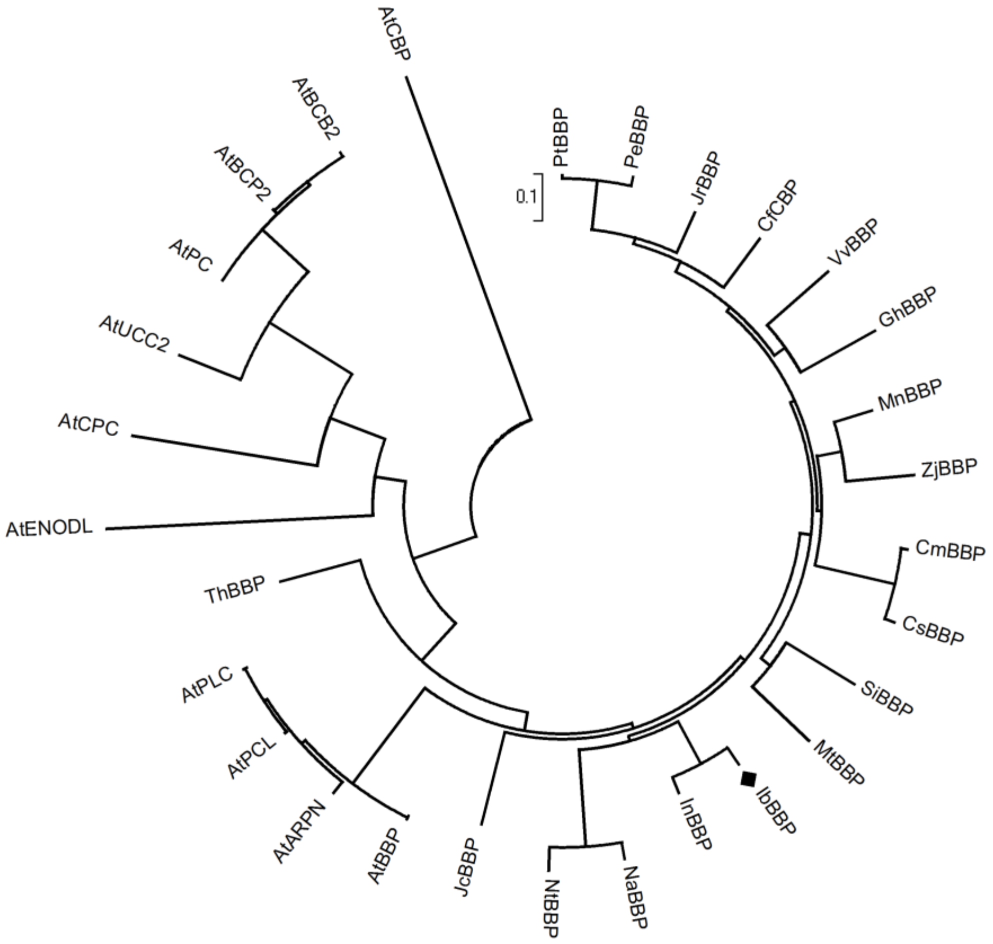


**Fig. S5. Phylogenetic tree of basic blue protein (BBP) family in plants.**

Phylogenetic analyses of the BBP family in *Ipomoea batatas* (Ib), *Arabidopsis thaliana* (At), *Ipomoea nil* (In), *Juglans regia* (Jr), *Nicotiana tomentosiformis* (Nt), *Nicotiana attenuate* (Na), *Sesamum indicum* (Si), *Medicago truncatula* (Mt), *Jatropha curcas* (Jc), *Populus euphratica* (Pe), *Populus trichocarpa* (Pt), *Morus notabilis* (Mn), *Cucumis melo* (Cm), *Cucumis sativus* (Cs), *Tarenaya hassleriana* (Th), *Cephalotus follicularis* (Cf), *Ziziphus jujuba* (Zj), *Vitis vinifera* (Vv) and *Gossypium hirsutum* (Gh) are shown. Alignment of the complete protein sequences of BBP family was carried out by ClustalX2. The phylogenetic relationships were analyzed by the neighbor-joining method. A bootstrap analysis of 1000 resampling replications was conducted in MEGA 4.1.

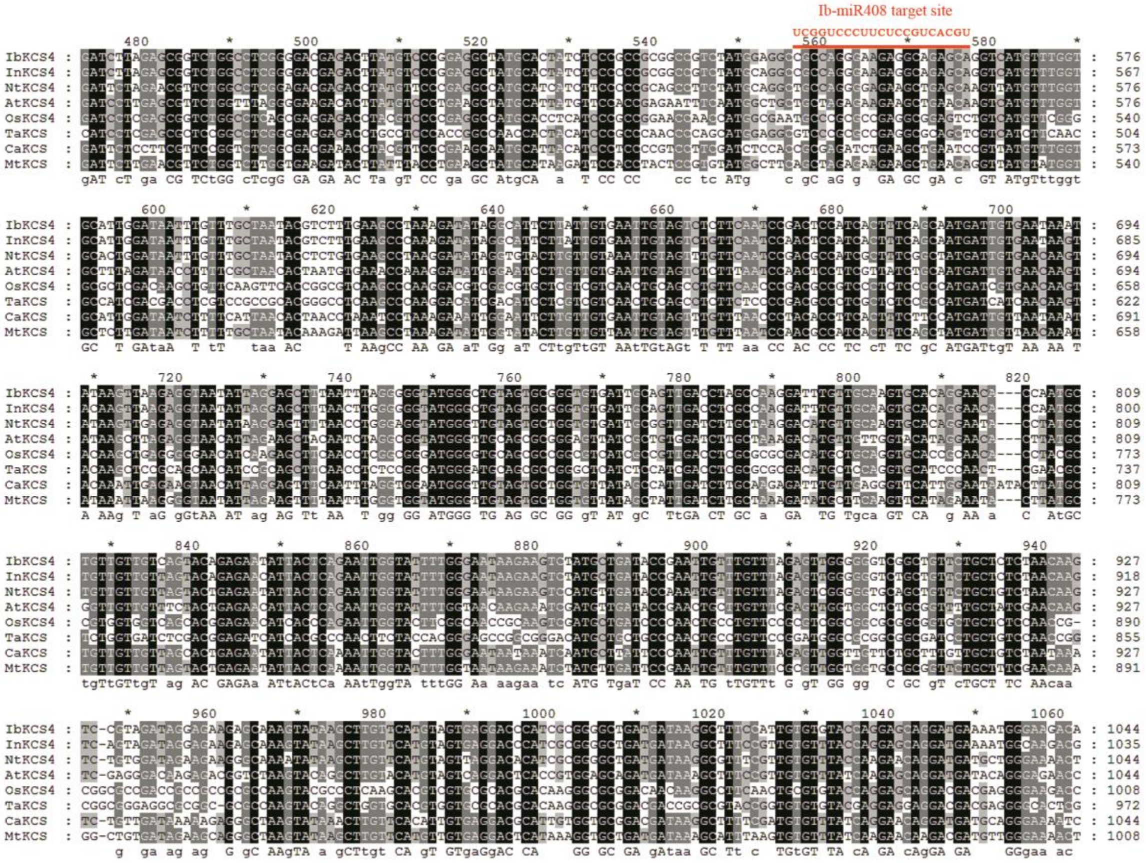


**Fig. S6. Comparisons of *3-ketoacyl-CoA synthase-like gene* (*KCS*) in plants.**

Comparisons of the *KCS* sequences among *Ipomoea batatas* (Ib), *Ipomoea nil* (In), *Nicotiana tabacum* (Nt), *Arabidopsis thaliana* (At), *Oryza sativa* (Os), *Triticum aestivum* (Ta), *Cicer arietinum* (Ca) and *Medicago truncatula* (Mt) are shown. The red underline indicates the Ib-miR408 binding site. The identical nucleotides are shaded by black color, and the similar nucleotides are shaded by gray.

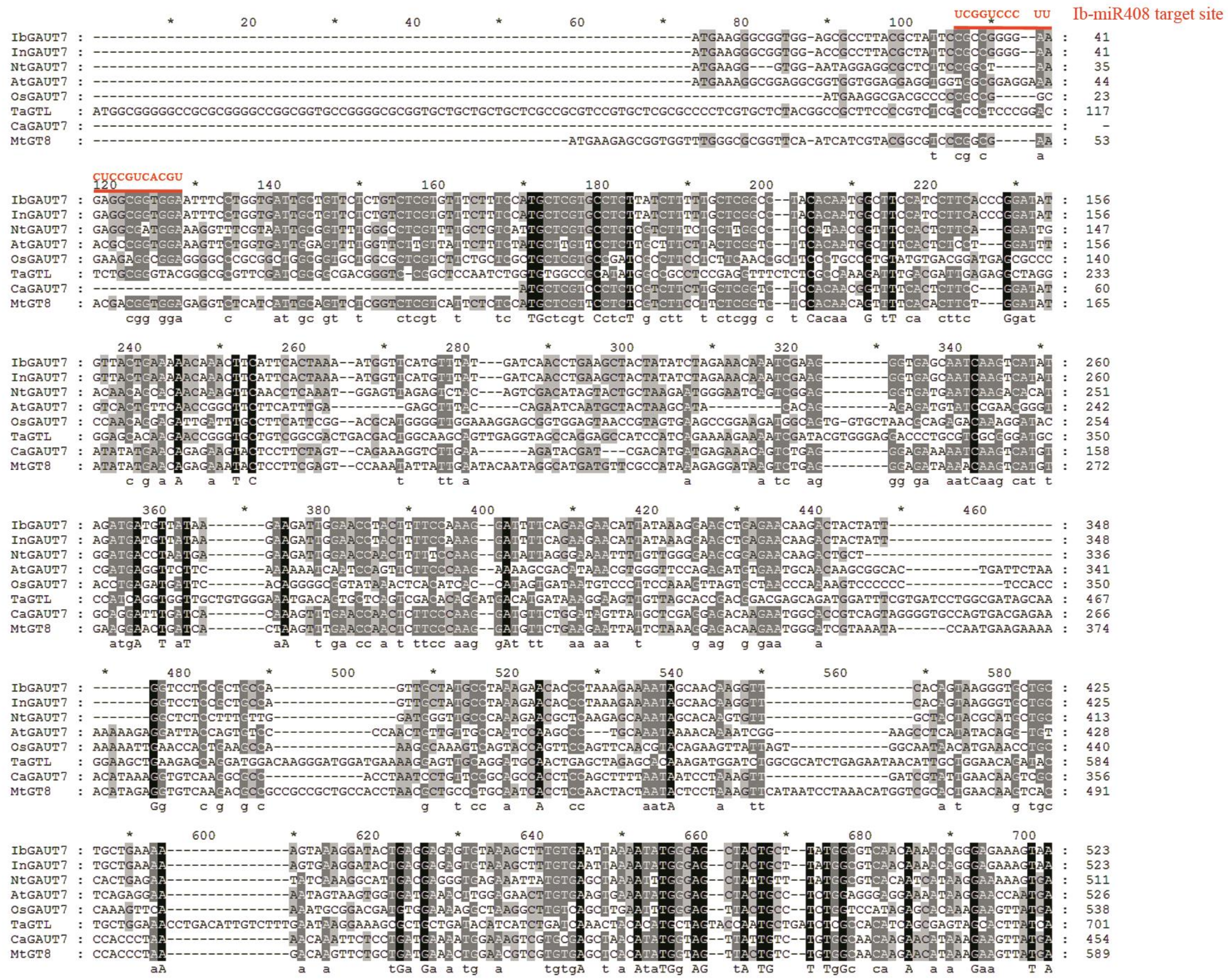


**Fig. S7. Comparisons of *galacturonosyltransferase-like* gene (*GAUT*) in plants.**

The comparisons of *GAUT* sequence among *Ipomoea batatas* (Ib), *Ipomoea nil* (In), *Nicotiana tabacum* (Nt), *Arabidopsis thaliana* (At), *Oryza sativa* (Os), *Triticum aestivum* (Ta), *Cicer arietinum* (Ca) and *Medicago truncatula* (Mt) are shown. The red underline indicates the Ib-miR408 binding site. The identical nucleotides are shaded by black color, and the similar nucleotides are shaded by gray.

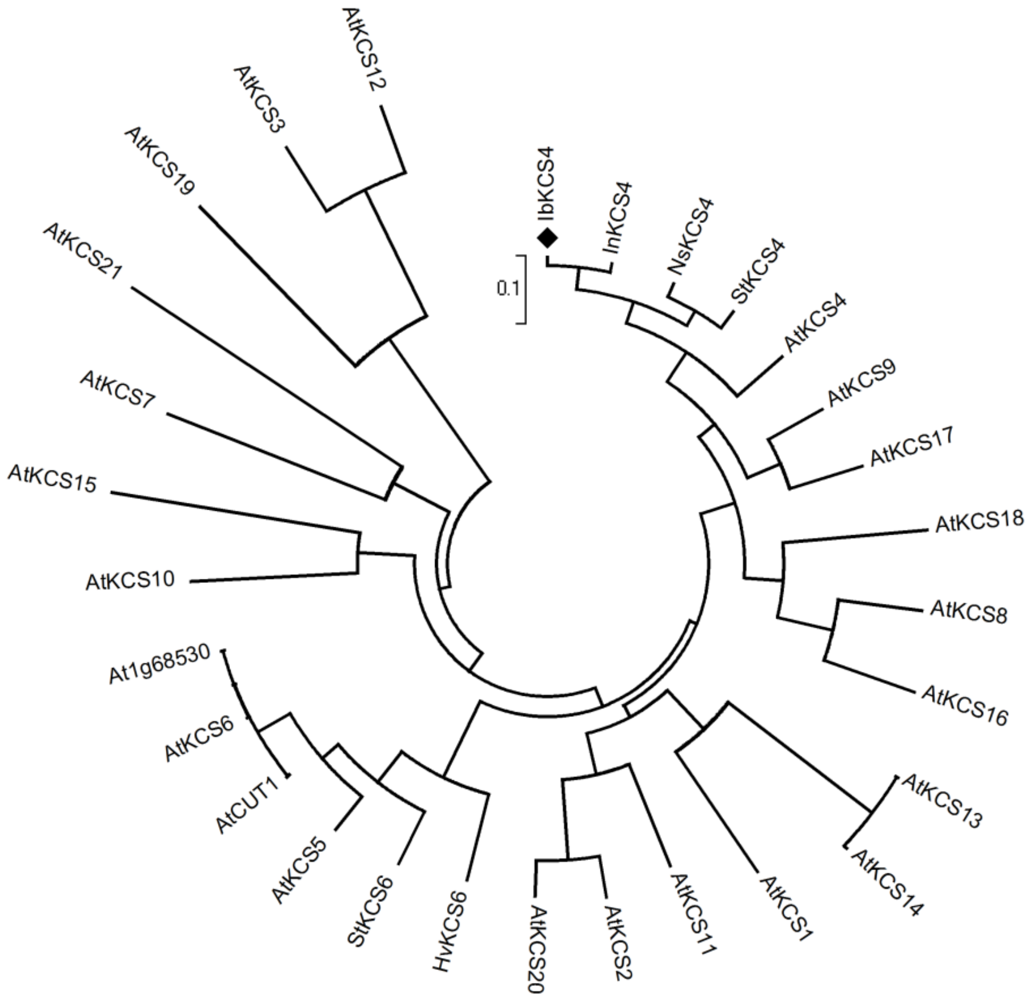


**Fig. S8. Phylogenetic tree of KCS protein family in plants.**

Phylogenetic analyses of the KCS Family in *Ipomoea batatas* (Ib), *Arabidopsis thaliana* (At), *Ipomoea nil* (In), *Solanum tuberosum* (St), *Nicotiana sylvestris* (Ns) and *Hordeum vulgare* (Hv) are shown. Alignment of the complete protein sequences of the KCS family was carried out by ClustalX2. The phylogenetic relationships were analyzed by the neighbor-joining method. A bootstrap analysis of 1000 resampling replications was conducted in MEGA 4.1.

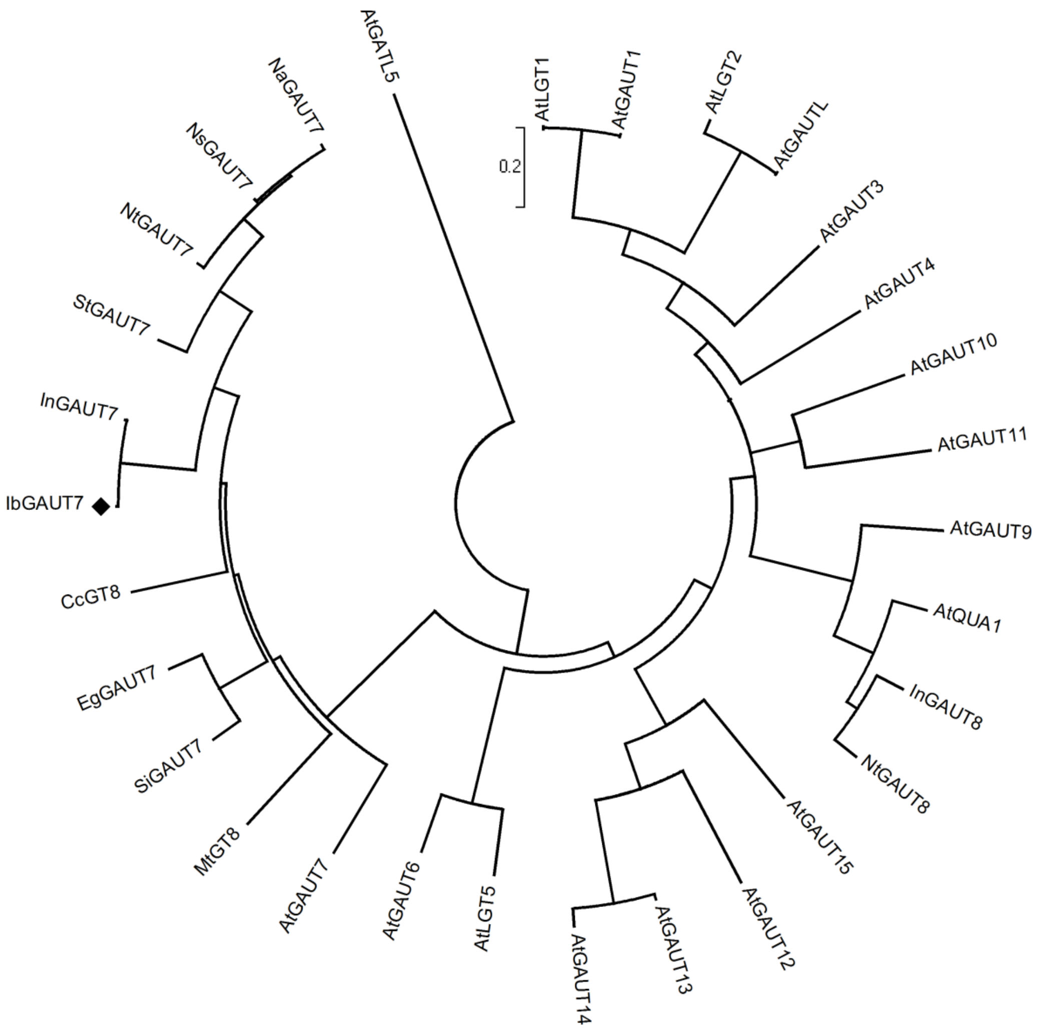


**Fig. S9. Phylogenetic tree of GAUT protein family in plants.**

Phylogenetic analyses of the GAUT Family in *Ipomoea batatas* (Ib), *Arabidopsis thaliana* (At), *Ipomoea nil* (In), *Solanum tuberosum* (St), *Nicotiana tomentosiformis* (Nt), *Nicotiana sylvestris* (Ns), *Nicotiana attenuate* (Na), *Sesamum indicum* (Si), *Erythranthe guttata* (Eg), *Cynara cardunculus var. scolymus* (Cc) and *Medicago truncatula* (Mt) are shown. Alignment of the complete protein sequences of the GAUT family was carried out by ClustalX2. The phylogenetic relationships were analyzed by the neighbor-joining method. A bootstrap analysis of 1000 resampling replications was conducted in MEGA 4.1.
